# *Ab Initio* Coordination Chemistry for Nickel Chelation Motifs

**DOI:** 10.1371/journal.pone.0126787

**Published:** 2015-05-18

**Authors:** R. Jesu Jaya Sudan, J. Lesitha Jeeva Kumari, C. Sudandiradoss

**Affiliations:** Bioinformatics Division, SBST, VIT University, Vellore, 632 014, India; University of Rome Tor Vergata, ITALY

## Abstract

Chelation therapy is one of the most appreciated methods in the treatment of metal induced disease predisposition. Coordination chemistry provides a way to understand metal association in biological structures. In this work we have implemented coordination chemistry to study nickel coordination due to its high impact in industrial usage and thereby health consequences. This paper reports the analysis of nickel coordination from a large dataset of nickel bound structures and sequences. Coordination patterns predicted from the structures are reported in terms of donors, chelate length, coordination number, chelate geometry, structural fold and architecture. The analysis revealed histidine as the most favored residue in nickel coordination. The most common chelates identified were histidine based namely HHH, HDH, HEH and HH spaced at specific intervals. Though a maximum coordination number of 8 was observed, the presence of a single protein donor was noted to be mandatory in nickel coordination. The coordination pattern did not reveal any specific fold, nevertheless we report preferable residue spacing for specific structural architecture. In contrast, the analysis of nickel binding proteins from bacterial and archeal species revealed no common coordination patterns. Nickel binding sequence motifs were noted to be organism specific and protein class specific. As a result we identified about 13 signatures derived from 13 classes of nickel binding proteins. The specifications on nickel coordination presented in this paper will prove beneficial for developing better chelation strategies.

## Introduction

Metals are important constituents of life, driving economic activity and industry [1]. The increased usage of heavy metals in modern industries leads to an increase in the environmental stress. Nickel and its compounds represent a good example among metals with extensive industry usage. Nickel compounds are released to the environment through the acceleration in utilization of nickel-containing products at all stages. Therefore accumulation of nickel in the environment may lead to serious human health hazards [2, 3]. Noxiousness of nickel and its compounds was mediated to universal and occupational inhabitants via air, water and food. Nickel intake routes for humans are dietary ingestion, inhalation and dermal contact. Inhalation is an imperative route of exposure to nickel in relation to health risk. Toxic effects depend on the chemical species, their physical form as well as on their concentration and exposure pathway [4, 5]. Hazards of nickel exposure have been reported to include skin allergy, delayed hypersensitivity, delayed pulmonary symptoms, bronchitis, asthma, nonmalignant respiratory disorder, immunotoxicity, genotoxicity, gene silencing, carcinogenicity, fetal and embryo toxicity [6–14]. Nickel toxicity occurs on the basis of the following four mechanisms (a) replacement of essential metal by nickel in metalloproteins, (b) association of nickel with catalytic residues of non-metalloenzymes, (c) allosteric inhibition of an enzyme by binding outside the catalytic site (d) nickel induced oxidative stress [15].

Removal of toxic metal from the biological inhabitants involves different methods of chelation that employs synthetic, chemical and peptide chelators. Simple coordination chemistry resolves the perception of metal chelation. Therefore investigating the association of toxic metal with functionally vital macromolecules can produce significant results on the type, geometry and structure of residues that favors such coordination. These results when combined with chelation therapy produce new insights into designing effective peptide chelators. Many studies have employed coordination chemistry in designing effective chelators for toxic metal chelation [16–19]. Our earlier work on arsenic, beryllium, cadmium and lead endow with valuable information in chelate patterns and geometry that can be employed in their chelation [20, 21]. The present study is aimed to determine the coordination geometry of nickel, in a view of understanding the association of nickel carcinogenicity in biological molecules and to support chelator designing. The investigation of nickel coordination was carried out on the basis of the work describing geometry of metal ion-binding sites within proteins by Lumbomir Rulisek (1998) [22] and Harding M.M. (2004) [23]. This study gives an account of the residue, residue position, donor atom and distance that gives an insight on the choice of chelators. We also examined the distribution of bond lengths, coordination numbers, B-factor (displacement parameter sometimes referred as ‘temperature factor’) and relative occupancies of metal ions [24].

## Result and Discussion

### Validation by B-factors of the metal ion and coordinating residues

As an initial step in dataset validation, all 321 nickel bound structures were subjected to nickel coordination analysis. Subsequently, the B-factors and occupancies of residues within 3Å of nickel environment were predicted, as all atoms within 3Å of a metal ion were considered to be interacting atoms [24]. The observed individual and mean B-factors of the coordinating residues were noted to be >0.2Å^2^ and occupancies were within the stable range of 0.5–1.0. Since the B-factors of metal should be closer to the B-factor of its environment, all outliers exceeding an average deviation of ≥7.0 Å were ignored to obtain a statistically significant correlation of 0.83 (S1 Fig) [24]. The above method helped us to filter down the dataset to 186 diverse nickel bound structures which were subjected to further analysis.

### Nickel-coordinating residues

**Fig 1:**
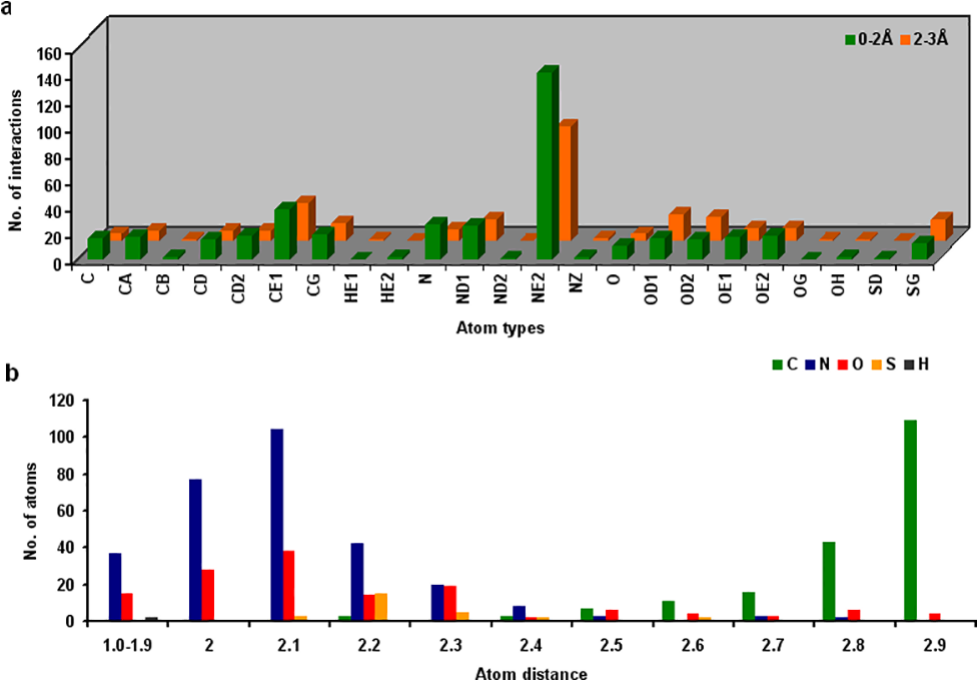
Atom preferences in nickel coordination. Figure represents a) the various atom types coordinating with the Nickel and b) shows the interactions distances of atoms with the metal.

Similar to most metals, the amino acids namely histidine, aspartic acid, glutamic acid and cysteine were the most favored residues to coordinate nickel, among which, histidine was noted to dominate (p-value = <0.0001) over other amino acids (S2 Fig). The non-polar residues like phenylalanine, leucine and isoleucine as well as less polar amino acids proline and threonine showed no preference for nickel coordination. Other hydrophobic amino acids namely methionine, valine, tryptophan, polar amino acids arginine, glutamine, asparagine, serine and less polar amino acids glycine, tyrosine, alanine were observed coordinating with nickel in an insignificant manner. The atomic preference among the nickel coordinating residues within the cut-off distance (3Å) was predicted and a comparison of side-chain and backbone atoms was made. Fig 1A illustrates the atoms of coordinating residues and their subsequent number of interactions with nickel. Since histidine residue plays a dominant role in nickel coordination it was apparent that ε-nitrogen (NE2) of its imidazole ring had the maximum number (p-value = 0.0001) of coordination (S3 Fig). Nevertheless, the ε-carbon (CE1) was noted to be the next dominant, however not due to its coordination with the metal, but because of its proximity to the ε-nitrogen group. The positive charge on nitrogen groups in the imidazole ring makes it a prominent nucleophilic center in coordinating with metal. The other backbone atoms of histidine residue had negligible association with the metal. Apart from the amino groups, the side-chain carbonyl groups of aspartic acid and glutamic acid also coordinated with the metal. The backbone atoms of these residues were masked for the metal coordination due to the increased hydrophilicity at the side-chain atoms. Conversely, the amide structures of glutamic acid and aspartic acid namely glutamine and asparagine showed coordination through backbone and side-chain atoms. Sulphydryl groups of cysteine were also equally preferred for nickel coordination, though, were observed only in limited number of entries. Similar to aspartic acid and glutamic acid no backbone coordination was observed with cysteine residues. On the contrary, residues namely tryptophan, methionine, valine, serine, arginine, glutamine, asparagine, glycine, tyrosine and alanine did show backbone coordination especially at the alpha carbon (Cα). On the whole, the study revealed that nickel has a higher preference for side-chain atoms over backbone atoms. Of the 186 structures, about 153 structures had histidine as one of the coordinating residues within the cut-off distance among which, “Histidine-only” chelates accounted for 78 structures. The other prominent coordinating residues namely aspartic acid and glutamic acid were noted in about 40 and 32 structures respectively. Cysteine based chelates were limited to 18 structures. Due to the presence of several atom types within the coordination space, metal atom distances were calculated to justify atom association with the metal. From the investigation, we identified nine atoms namely NE2, HE2, ND1 of histidine, OD1, OD2 of aspartic acid, OE1, OE2 of glutamic acid, N of serine, histidine, glycine, cysteine and OD1 of asparagine at distances of <2 Å from the metal. The distance ranges for all the atom types varied between 1.2 and 3.0Å. The minimum and maximum distance range observed for nitrogen atoms were 1.8Å and 2.9 Å, for oxygen 1.6 Å and 2.9 Å, for carbons 2.1 Å and 3.0 Å and for sulfur 2.0 Å to 2.7 Å and is plotted in Fig 1B. From the study, we found that the presence of at least one of the atoms either from histidine (NE, ND) or from aspartic acid / glutamic acid (OD/OE) is required to coordinate with nickel ion. These distances were however validated by comparing the average distance for each atom type of high resolution (0–2.0Å) with medium resolution (2.0–3.0Å) structures. The correlation plot (S4 Fig) obtained revealed a positive correlation of 0.945.

### Nickel coordination geometry

The chelate loops represented by the coordinating residues were predicted for all structures and are listed in S1 Table. The coordination table lists (S1 Table) for each structure, the chelate loop and its size, positional spacing of residues within the chelate, the residue length of the chelate, number of heteroatoms coordinating the metal and coordination number (cn). Additionally, the secondary structural features of the chelate-loop residues and the structural classification of the proteins according to CATH and SCOP are provided [25]. The chelate patterns and their similarity and dissimilarity among identical and distinct protein structures were analysed and are discussed.

### Chelate-loop patterns

Unlike cadmium, beryllium, arsenic and lead, the chelates of nickel were very similar even among non-identical structures [20, 21]. The maximum number of protein donor atoms coordinating the metal was noted to be 5 (PDB ID: 3CGM, 2Y39). The maximum coordination number (cn) reached upto 8 (PDB ID: 3QC3) indicating the preference for even a larger group of atoms. However it was observed that, increase in cn was associated with the presence of heteroatoms including solvent atoms. In structures with cn 1, a single histidine was effective enough to coordinate with the metal. However without a clear knowledge of the proximal residues it would be naive to arrive at this conclusion, because of all the structures analyzed, about 95% have cn>1. In contrast to arsenic, beryllium, lead and cadmium bound structures [20, 21], coordination with nickel require the presence of at least a single residue donor within the coordination sphere (3Å), despite the presence of many solvent and non-residue donors. This proves the advantage and thereby a need for a biological chelator over chemical chelators. The chelate patterns did not vary much and the most common donor pairs are given in Table 1A. Among all donor pairs, the HH patterns (histidine-histidine) were predominant and co-occur with acidic residues namely aspartic acid and glutamic acid. Single residue and tri-peptide chelators had the maximum number of occurrences (Table 1B **)**, however the chelate sizes, which gives the distance between the terminal residues of a chelate varied drastically over all structures. The chelate sizes ranged between 1 and 550 (Table 1C), indicating the importance of precise protein folding to place the distantly positioned residue proximal to the metal. Strikingly, identical chelates also varied in length, as observed in 1J6P and 3IAR structures (**S1** Table) but were noted to belong to the same structural fold. However this was observed only with larger chelates (≥4 donors), wherein with smaller chelates, the structural folds, chelate sizes and residue position varied as evident from HHH chelates (S1 Table). Also the donor distribution or spacing within a chelate was noted to be random, yet among “Histidine only” chelates, successive histidines were noted at 2^nd^ and 4^th^ positions in most structures. Also in histidine and aspartic acid chelates (HD), the residues were placed alternatively as seen in PDB IDs 3II2, 1S3Z, 4DIQ etc. No specific positional arrangements were noted in histidine-glutamic acid chelates, however the glutamic acids were noted to be within 10 residue length from histidine. In glutamic acid-glutamic acid (EE) chelates, donor spacing of 3 and in aspartic acid-aspartic acid (DD) chelates residue spacing of 2 were preferred. In case of “Cysteine only” chelates, the cysteine co-occurrence was noted to be spaced at 3 or 4 residue length. The co-occurrence pattern for the coordinating residues were predicted using mutual information analysis (S5 Fig), which indicate histidine, glutamic acid and aspartic acid to occur in combination, while cysteine residues were negligible due to minimum number of entries. The sequence logo patterns show the nickel binding site to be populated by hydrophilic amino acids [26]. We noted that with small identical chelates, the donor positioning followed a similar pattern but vary in their structural folds. On the contrary, large but identical chelates show different donor spacing yet represent identical folds. The analysis reports that nickel coordination is purely random with no specificity towards folds or sequence patterns. However, the chelate length plays a major role, as the fold and residue positioning is dependent on the length with histidine being the most favored residue [27].

**Table 1:** 

**a. Predominant donor pairs and its occurrence in dataset entries**													
Donor pairs	AH	CC	CG	CH	DD	DH	EE	EH	GS	HD	HE	HH	HK
No. of occurrences	2	3	2	5	6	7	4	3	4	16	13	48	3
**b. No. of minimum and maximum donors within first shell proximity to nickel**													
No. of donors	0		1		2		3		4		5		
No. of chelates	0		61		44		62		17		2		
**c. Diverse ranges of chelate lengths**													
Chelate size	0	1	2	3	4	5	6–20	21–50	51–100	101–200	201–400	401–550	
No. of chelates	0	61	4	14	7	5	15	25	34	16	8	2	

The table represents a) the donor pairs that are observed in at least 2 structures deciphered from the coordination table (S1 Table) b) The number of donors and chelates were obtained from the coordination table (S1 Table) c) Chelate size refers to the distance between the first and the last residue of a chelate. The number of chelates within a specific range was calculated from the coordination table (S1 Table)

### Secondary structural conservation of donor residues

The secondary structural stability of chelate residues was analyzed by predicting the distribution of φ-ψ angles across the five regions of Ramachandran plot. The five regions were classified as α-helix (φ = -180 to 0°, ψ = -120 to 60°) constituting the region A, β-sheet (φ = -180 to 0°, ψ = 60 to -240°) representing the region B and left-handed α-helix (φ = 90 to 100°, ψ = -20 to 80°) corresponding to E, while C and D refer to the partially allowed region φ = -180 to -40°, ψ = 0 to -40° [28]. The torsion angles for all the chelate residues were predicted using SwissPDBviewer [29] and the Ramachandran plot for the residues (S6 Fig) show the helices to be well confined to region A, however the chelate residues of the sheets were not very stable. This is inferred to be due to the stabilized intra-residue hydrogen bonding within helices. Nevertheless, the turns/coils were the other dominant structural pattern observed among helices.

### Geometries involved in nickel coordination

**Fig 2:**
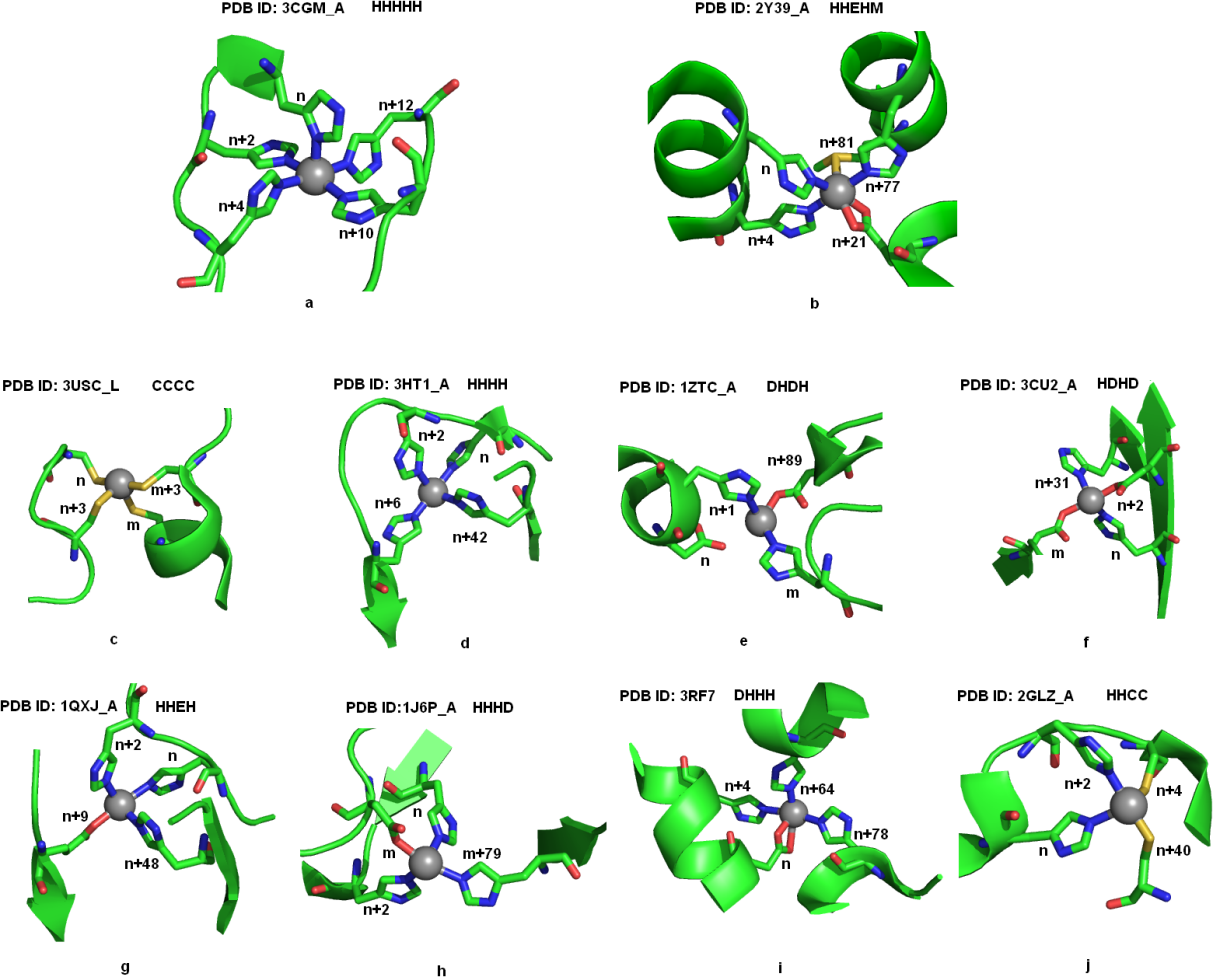
Coordination geometries in penta and tetra-residue chelates. Fig 2a and 2b show the penta-residue coordination towards Nickel displaying a square pyramidal geometry with HHHHH chelate and an octahedral geometry with HHEHM chelate. The bi-dentate coordination of glutamic acid at n+ 21 positions is observed through the coordination of its ε- oxygen atoms. Fig 2c-2j displays the tetra- peptide coordination of nickel. Residue positions are labeled in the figure where, ‘n’ represents the position of the first residue in the chelate and the following number indicates the distance of successive residues relative to the initial residue of the chelate. The letter ‘m’ denotes the position of a residue if separated by a distance >100 residues. The coordinating residues are displayed as sticks and as cartoon to highlight the secondary structures. The atom coloring represents carbon (green), nitrogen (blue), oxygen (red) and sulphur (yellow). Nickel atom is shown as sphere and colored in grey.

**Fig 3:**
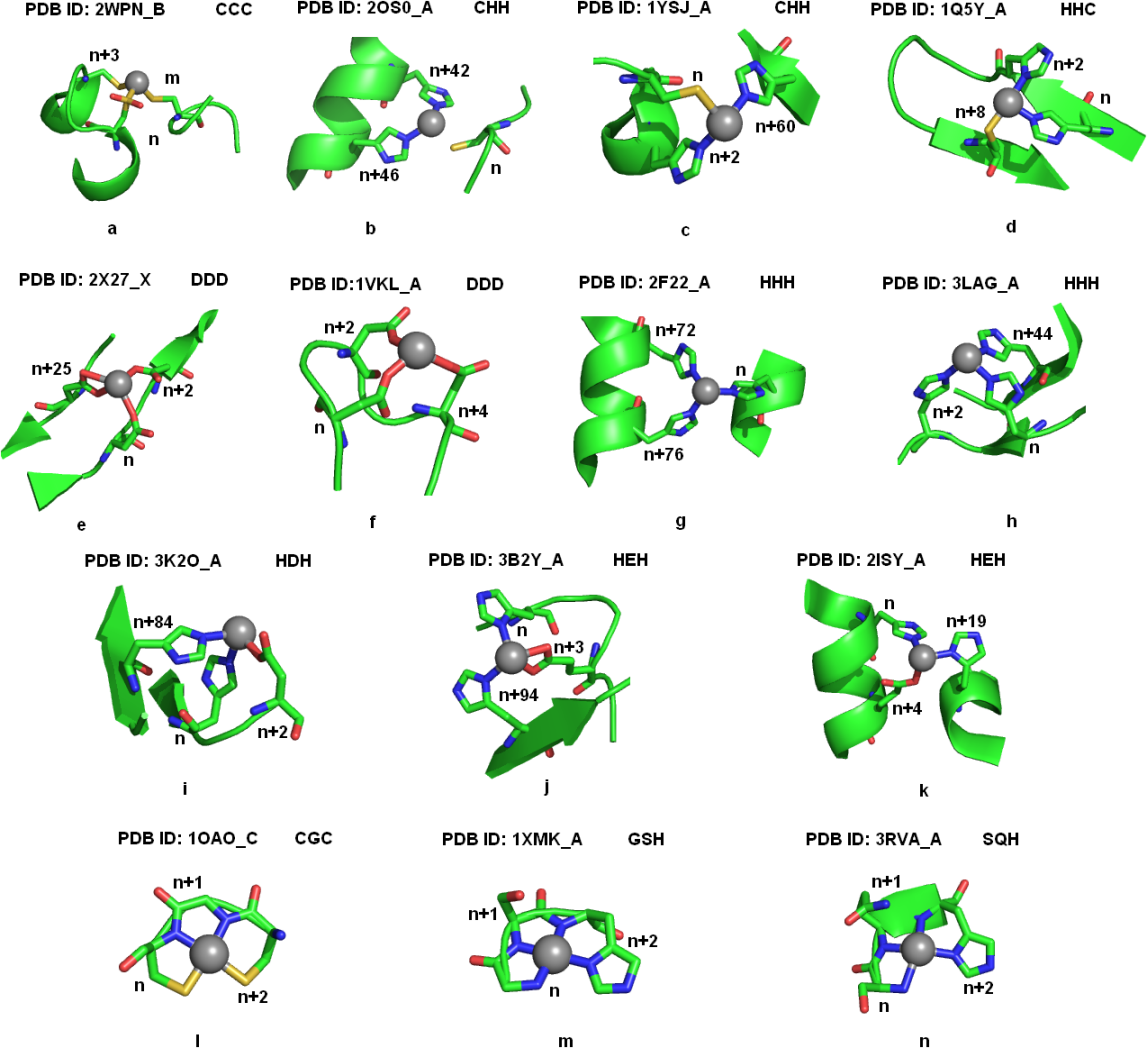
Coordination geometries in tri-residue chelates. The figure shows the dominating coordinating patterns among the tri-residue chelates. Fig 3a-3d shows cysteine based chelates. Fig 3e and 3f shows the different geometries among DDD chelates. Fig 3g and 3h display the secondary structural differences among HHH chelates. Fig 3i, 3j and 3k represent the coordination of aspartic acid and glutamic acid in association with histidine. Fig 3l, 3m, and 3n show the identical coordination pattern of residues spaced at a distance of 1 residue length. Residue positions are labeled in the figure where, ‘n’ represents the position of the first residue in the chelate and the following number indicates the distance of successive residues relative to the initial residue of the chelate. The letter ‘m’ denotes the position of a residue if separated by a distance >100 residues. Coordinating residues are displayed as sticks and as cartoon to highlight the secondary structures. The atom coloring represents carbon (green), nitrogen (blue), oxygen (red) and sulphur (yellow). Nickel atom is shown as sphere and colored in grey.

**Fig 4:**
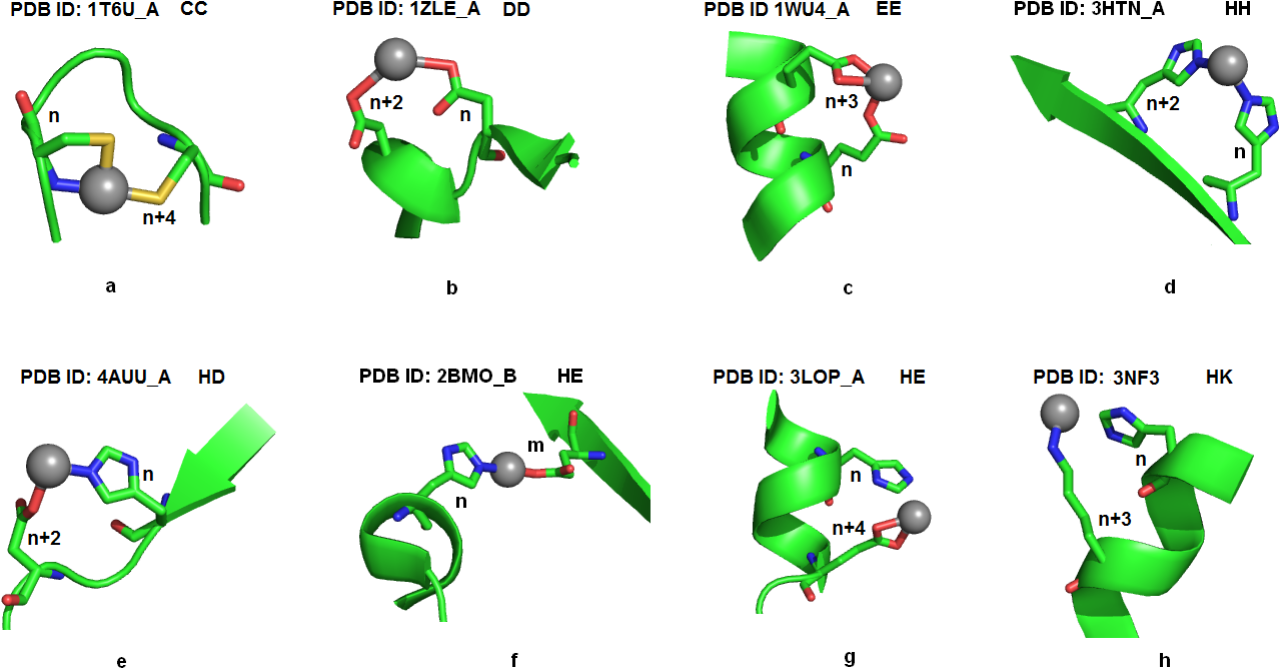
Coordination geometries in bi-residue chelates. The figure shows the dominating patterns among the bi-residue chelate. Residue positions are labeled in the figure where, ‘n’ represents the position of the first residue in the chelate and the following number indicates the distance of successive residues relative to the initial residue of the chelate. The letter ‘m’ denotes the position of a residue if separated by a distance >100 residues. Coordinating residues are displayed as sticks and the atom coloring represents carbon (green), nitrogen (blue), oxygen (red) and sulphur (yellow). Nickel atom is shown as sphere and colored in grey.

All nickel coordination geometries for bi, tri, tetra and penta residue chelates were observed using Pymol molecular graphics system [30]. Figs 2, 3 and 4 displays the pictorial representations of penta, tetra, tri and bi-peptide chelate geometries involved in nickel coordination, observed in high resolution structures. The coordinating geometries in nickel coordination were observed to be donor dependent. With penta-peptide chelates a square pyramidal geometry was observed and through the bi-dentate coordination of glutamic acid, an octahedral geometry was obtained (Fig 2A and 2B). In case of tetra-peptide chelates, tetrahedral geometry was observed in CCCC (Fig 2C) and HHCC (Fig 2J), whereas the other patterns amely HHHH (Fig 2D), HDHD (Fig 2F), HHEH (Fig 2G) and HHHD (Fig 2H) displayed disphenoidal geometry. **S6A** Fig displays the superimposed disphenoidal geometry of the commonest tetra-peptide chelates. The HHHH chelate exhibited perfect see-saw showing bond angles 94.04° and 176.76°, while other tetra-peptide chelates had a distorted geometry. All tripeptide chelates exhibited trigonal pyramidal geometry (Fig 3) except CHH and HCC which were trigonal planar (Fig 3C and 3D). When a bi-dentate coordination of aspartic acid or glutamic acid (Fig 3E and 3J) occurs, a tetrahedral geometry was observed among tripeptide chelates. The superimposed structures of the prominent tripeptide chelates displayed in S7C–S7E Fig showed the histidine-only chelates (S7E Fig) to be geometrically identical irrespective of the donor positions (S1 Table). Strikingly with bi-residue chelates, the expected linear geometry was least noted (Fig 4) unlike the CC chelates (Fig 4A), where the cysteine pair lie in plane to the nickel atom. In contrast, the EE chelates show a trigonal pyramidal geometry due to the bi-dentate coordination of glutamic acid (Fig 4C). The EE chelates show a random coordinating geometry depending on the proximity of the residues. Superimposition of EE chelates (S7F Fig) shows structural deviation despite the similarity in residue spacing. On the contrary, superimposition of HHH chelates (S7G Fig) appears to be structurally identical, though the residues were differently positioned. As seen in Figs 2, 3 and 4 the different geometries of chelates are characterized mostly by the number of donors, but rarely on the donor type. All the chelates were noted to exhibit either a mono-dentate or bi-dentate coordination with nickel through atleast one of its donor atoms. Among all donor types, histidine was observed to be the most common residue involved in atom-atom contact when the distance was <2.15Å. Structures with single and bi-residue chelate show mono-dentate coordination, with an exception of glycine (PDB ID: 1HYO), tryptophan (PDB ID: 2POS) and alanine (PDB ID: 1JVL, 1IA6, 1IA7) that shows bi-dentate coordination through its backbone, side-chain and both backbone and side-chain respectively. All residues proximal to glycine within a chelate, was noted to form a bi-dentate coordination as noted from CGC, GSH, GH and GS chelates. This implies the structural importance of glycine in providing flexibility to the chelate loop. In HEH tri-residue chelates, glutamic acid residue show bi-dentate coordination, nevertheless all aspartic acid were noted to prefer only mono-dentate coordination with nickel even in DDD chelates. Strikingly all histidines exhibit mono-dentate coordination in all single residue, bi-residue and tri-residue chelates. Interestingly, histidine when supplied as a ligand show tridentate coordination as observed with PDB ID 3RQT. This validates histidine as a potential peptide chelator in nickel chelation.

### Structural folds and secondary structure association of chelates

Proteins belonging to different structural classes were identified to coordinate with the metal. However the structural folds for about 48% of the nickel bound structures were not characterized. Some of the major classes of folds among nickel bound proteins included 3-layer sandwich, alpha-beta barrel, up and down bundle, ferredoxin fold, double stranded beta helix etc. No specific folds were associated with specific chelate, although identical chelates belonged to identical folds as observed with HHHD (Alpha-Beta Barrel), HHEH (Double-stranded beta-helix), and HDHD (Alpha-Beta Barrel) chelates.

Nickel associations with alpha helices were noted to be dominant among up and down bundle folds, in which the nickel atom was noted to coordinate with residues from all helices as shown in 2Y39 (Fig 2A) thereby stabilizing the helical architecture. Residues associated with β-sheets show diverse fold specificity, although β-barrel fold was common among bi-residue and tetra-residue chelates. In alpha-beta barrel fold, the nickel coordinating residues were mostly located at the start or end regions of the beta sheet and therefore does not represent a strand despite its presence closer to sheets (Fig 2D and 2H). Preference for specific structural folds was not observed in smaller chelates. For instance, the histidine triplet chelate (HHH) was noted to be characteristic of up-down bundle, double stranded beta helix and lipocalins structural folds, while CHH chelates were common for alpha-beta complex, peptide deformylase and phosphorylase/hydrolase like folds. As the chelate size reduced, the fold specificity also reduced. The other striking feature noted among smaller chelates was that, even when identical chelates share identical folds, the secondary structural features of the chelates varied. This can be inferred from the HEH chelate (PDB ID: 1J5Y, 2ISY, 3B2Y) with different secondary structural preferences, but not so with CHH, HHH chelates. Secondary structural preferences were noted only for chelates like GSH (turns), HHC (sheet) and EE (helix). However, based on the donor spacing within the chelates, we were able to envisage the secondary structural preferences and coordination patterns. In all the chelates when the coordinating residues are positioned at n, n+3 or n, n+4, they were noted to lie in a helix (Figs 2C, 3G and 4C) or turn (Figs 2C, 3F and 4A) architecture. When residues are much closer they represent a strand or coil (Figs 2F, 3D and 4D). When residues are distantly present they are observed to connect two secondary structures like helix-helix (Fig 2A), sheet-sheet (Fig 2F), sheet-helix (Figs 3C and 4F) or turns—sheet/helix (Fig 2C and 2E).

### Choice of donors and donor spacing in nickel chelation

Every nickel bound protein showed its own preference for chelate pattern despite the presence of other potential residues for chelation. On observing about ten residues upstream and downstream the nickel coordinating environment, we noted stretches of residues potential enough to coordinate with the metal. For instance, in the HHC chelates (PDB ID: 1Q5Y,3PHT,2BJ7), the preferred coordination pattern as observed from the structure was H-X-H-[MI]-[ND]-[HE]-[DH]-[DIV]-C, wherein the underlined residues represent the metal coordinating residues. As can be seen from the pattern there are many other potential patterns within the motif like H-X(2,3)-[H] that can coordinate with the metal. But, structurally these residues were noted to lie in a loop, and the geometry of the residues was conformationaly unfavorable to coordinate the metal as they were oppositely oriented. Similarly at the upstream of the pattern there were stretches of histidines (HHHD) representing helix architecture, however the residues were restrained from the metal as a result of its geometry. For histidine-cysteine chelate, no coordination was noted when the residues were immediately adjacent to each other. In the DDD chelate of 1VKL, the coordination pattern was noted to be D-G-D-G-D (Fig 3F). Whereas in the other DDD chelate (PDB ID 2X27) the donors had a different spacing (n, n+2, n+25). At the upstream of this pattern we noted another DDD motif with n, n+2, n+4 spacing similar to 1VKL, but surprisingly was not associated with the metal. This was observed due to the presence of Val residues within the pattern (D-V-D-V-D) which induces rigidity and therefore alters the geometry of the residues inhibiting its ability to chelate. In the DHH chelate (3LMW) we noted that despite stretches of histidine, only alternate histidines are involved in coordinating the metal. Similarly we noted in histidine-aspartic acid chelates the residues prefer either sheet or turns and no helixes were involved. In addition, the residue spacing between histidine-aspartic acid was dominant in n, n+2 positioning as observed in HDHD and HDH chelates (Figs 3I, 2F and 2H). Additively, in HEH chelates the preferred spacing for histidine-glutamic acid residues were noted to be n, n+3 (Fig 3J and 3K). This can be inferred from 3B2Ywhere, the HEH coordinates with n, n+3, and n+94 despite the presence of H-X-E (n, n+2) at its vicinity. The EE chelates also follow the same pattern for coordination when the residues are spaced in E-X(2)-E. We observed in the structures 3B2Y, 2ISY and 1WU4, regardless of an E-X-E motif, the glutamic acid residues had no coordination with the metal as they were oppositely oriented due to the helical turn, however when separated two residues apart E-X(2,3)-E the residues complete a helical turn and face the same direction, therefore it coordinates with the metal as seen in Fig 3J and 3K. If coordination occurs at n and n+1or n+2 spacing, the residues should be present in a turn (PDB ID: 3AVR). Strikingly we noted that in a chelate, when residues are spaced at n, n+1 and lie in a loop having atleast one of the most coordinating residues, they form a clear square planar geometry as seen in CGC, GSH and SQH coordination (Fig 3L, 3M and 3N). Single residue chelates mostly preferred turns.

The presence of hetero atoms within the coordination space did not show any specific preference for the chelates. Two extremities showing single residue chelate with no non-protein donors (1XU2, 2I2O, 2IWB) and tetra-peptide donors with maximum of four heteroatoms (3QC3) were observed within the coordination space. Conversely in the penta-peptide chelates no heteroatom coordination was observed. This indicates the independency of donor atoms to coordinate with the metal. These results further justify that nickel coordination requires the presence of appropriate residues at suitable spacing within the coordination space. The observation of different chelates reveal that nickel coordination is random irrespective of fold and secondary structure of the chelate residues, however, despite the presence of well coordinating residues in the vicinity of the metal, the geometry of residues is mandatory to coordinate with the metal. This accounts for a clear knowledge of secondary structure and a complementing spacing of the chelate residues.

### Probability of succeeding and preceding residues in chelate loops

As an aid in designing effective peptide chelators, the probability of an amino acid to be involved in nickel chelation was predicted. Since the cn of nickel was noted to reach a maximum of 8 (3QC3), we considered only chelate loops with donors spanning a length (n) 1<n ≤10. A manual observation of all chelates revealed the residues alanine, aspartic acid, cysteine, glutamic acid, glycine, histidine, arginine, serine, glutamine and lysine to be involved in nickel coordination within a length span of 10 residues in a chelate. With the target residue (donor) at 0^th^ position, the succeeding residues and their probabilities at their respective positions were calculated and are tabulated in Table 2. The probability of a donor at a specific position is given as the number of occurrence of the given donor divided by the total number of occurrences of all the donors. Investigation of nickel bound structures showed that the predominant amino acids (histidine, aspartic acid, glutamic acid and cysteine) mostly occurred in combination with each other, as shown in Table 1B, or as multiples of the same residue separated at a specific distance. From Table 2 it is evident that histidine following a histidine at 2^nd^ position is the most favored with a probability of 0.185 and histidine at the 4^th^ position (0.096) is the next preferred spacing. Histidine with aspartic acid (0.083) at the 2^nd^ position was noted to be the third most dominant. Based on all the chelates specified in S1 Table, the probable succeeding and preceding amino acids in nickel chelation was observed and is given in Table 3. In “histidine-only” chelates successive histidine can occur at all positions except at the 7^th^ position in the ten residue length (Table 3). This indicates that stretches of histidine to as much as 6 residues are enough to coordinate the metal. However, we noted that even in a continuous stretch of His, nickel coordinates with alternative histidine or with distant histidine rather than the immediate residue. Similarly from Table 3, successive aspartic acid was noted at positions 2,3,4,6 and 9 whereas, successive glutamic acid was observed only in the 3^rd^ position. Successive cysteine residues were noted for a stretch of 4 residues or alternatively at positions 2 and 4. Aspartic acid in combination with histidine was observed in many structures and had a uniform positional spacing having aspartic acid at the 2^nd^ position from histidine, but not vice versa. Histidine residues were noted at 1^st^, 4^th^, 5^th^ and 7^th^ positions from aspartic acid. Glutamic acid and histidine were separated by 3 or 4 residues and histidine was noted at positions 2, 3 and 4 within the ten residue length span. Residue preferences for other amino acids were also noted but with low confidence. Although the probabilities of other residues were very minimal, the central point was that these residues if positioned at the right place would be capable of chelating nickel. From Table 3, several chelate patterns could be designed and some of the potential patterns deciphered from the table include H-D-H-X-H, C-C-X-C, D-X(3)-H, E-X(2,3)-H etc. Other sub-patterns include H-X(2,3)-K, S-Q-H and A-X(2,3)-H.

**Table 2:** Probabilities of amino acid occurrence.

First residue	Position of succeeding residues										Succeeding residues
	1	2	3	4	5	6	7	8	9	10	
**ALA**			0.006	0.006							**HIS**
**ASP**		**0.032**	0.006	0.006		0.006			0.006		**ASP**
	0.006			**0.012**	**0.012**		0.006				**HIS**
								0.006			**LYS**
**CYS**	0.012	0.012	**0.019**	0.012							**CYS**
	**0.019**	0.006									**GLY**
		0.006					0.006				**HIS**
**GLU**		0.006									**ASP**
			**0.012**								**GLU**
		0.006	0.006	**0.019**							**HIS**
**GLY**	0.006										**CYS**
	0.006	**0.019**									**HIS**
	0.025										**SER**
**HIS**	0.006	**0.083**							0.006		**ASP**
	0.006	0.006		0.006	0.006	**0.019**		**0.012**			**CYS**
		**0.019**	0.006	**0.025**		**0.012**	0.006	0.006	0.006		**GLU**
	**0.019**	**0.185**	**0.012**	**0.096**	**0.019**	**0.032**		0.006	0.006	**0.012**	**HIS**
**ASN**				0.006							**HIS**
**SER**	0.006										**GLN**
		0.006				0.006					**HIS**

The probabilities of residues at a specific position following an amino acid are displayed. Residues with high probabilities are highlighted in bold. The probability of a residue to occur at a specific position succeeding a donor is estimated as its total number of occurrences at that position divided by the total number of occurrences of all the donor residues.

**Table 3:** Putative amino acid positions for nickel coordination.

Positions										
0	1	2	3	4	5	6	7	8	9	10
**ALA**			H	H						
**ASP**	H	D	D	HD	H	D	H	K	D	
**CYS**	CG	CGH	C	C			H			
**GLU**		DH	EH	H						
**GLY**	HSC	H								
**HIS**	HSDC	DCEH	EHK	CEHK	HC	CEH	E	CEH	EHD	H
**ASN**				H						
**SER**	Q	H				H				

The table shows the probable combination of amino-acid occurrences for a 10 amino acid residue length within a 3Åcutoff distance from Nickel atom. The order and position of the residues were obtained from nickel chelates given in coordination table (S1 Table). Amino acids that do not coordinate with nickel are not shown in the table.

### Conserved sequence motifs of nickel binding proteins

Metal binding patterns were predicted from the conserved regions of nickel binding protein sequences retrieved from UniProtKB/Swiss-Prot database. No prominent similarity was observed among the sequences. Therefore the sequences were categorized according to their function and the domains specific patterns were predicted. The sequences belonged to a wide range of bacterial and archeal species that included actinomycetales, aquificales, bacteroidetes, chloroflexi, clostridia, cyanobacteria, deinococcus, dictyoglomus, gemmatimonas, methylacidiphilum, proteobacteria and solibacter, euryarchaeota and thermoprotei. The multiple sequence alignment of sequences within a family revealed good similarity, nevertheless we noted that even among identical strains sequence alignment showed a variation >50%, which accounts for sub-strain differences. However to get meaningful results we culled the sequences such that no two sequences shared similarity >90%. This was done to reduce redundancy among sequences. Table 4 shows the protein names and number of entries in each group considered in the study. Plausible nickel binding motifs among the sequences were identified based on the occurrence probabilities as given in Table 3 by checking for succeeding and preceding amino acids in the conserved regions of multiple sequence alignment. From the multiple sequence alignment several motifs were identified with that represented structural patterns, and therefore were considered to be potential enough to bind nickel. The conserved regions observed across the entire families showed the hydrophobic residues namely alanine, valine and leucine to be conserved and atleast one of the glycine were well conserved in all the species. In most cases, the presence of a conserved histidine was accompanied by a proline or glycine in its proximity indicating a preference for a structural arrangement either in a loop or turn. In view of the structural motifs, it’s seen that histidine dominate in coordinating the metal followed by aspartic acid and glutamic acid (Table 2). Accordingly, some set of sequences showed conservation of histidine especially among the bacterial group. The protein nickel/cobal efflux system showed stretches of conserved histidine and several short length motifs viz HHHH, HEHD, HDHD, DHHH and EHH were noted to be predominant. Nickel binding preference was noted to be highly selective even among related protein families. For instance, the structural motifs predicted for the nickel import ATP binding protein NikD and NikE were completely different and potential patterns predicted were G-S (NikD) and E-X(3)-H (NikE) according to the probability of occurrence (Table 2). However, most proteins like nickel responsive regulator, high affinity nickel transport protein, nickel ABC transporter show patterns with conserved histidine but in co-occurrence with other conserved residues like aspartic acid, glutamic acid, glycine etc. The positional arrangements of these conserved residues were in good agreement with the structural chelate residue spacing observed from structures and therefore were regarded as highly potential in binding nickel. Unlike other nickel binding proteins, the hydrogenase nickel incorporation proteins were conserved in cysteine residues with the patterns C-X(1,2)-[HC], C-X(2)-C, C-G-C showing high potential to bind nickel. This pattern was in par with the cysteine structural motifs predicted from structures 1T6U, 2WPN and 3USC.

**Table 4:** Probable nickel binding motifs from group of bacterial and archeal species.

Si. No.	Nickel binding proteins	Nickel binding patterns
1	Nickel-cobalt-cadmium resistance protein (NccB, CnrB, CnrC) (10)	[QEG]-**R**-[RKQK]
2	Hydrogenase nickel incorporation protein (HypA) (33)	**C**-X(1,2)-[**HC**], **C**-X(2)-**C**
3	Hydrogenase isoenzymes nickel incorporation protein (HypB) (8)	**C**-[TV]-[TVI]-**C**-G-**C**
4	Hydrogenase/urease nickel incorporation protein (HypB) (11)	**G-S**
5	Nickel import ATP-binding protein (NikD) (16)	**G-S**
6	Nickel import ATP-binding protein (NikE) (14)	**E**-X(3)-**H**
7	Nickel transport protein (NikQ) (4)	**D, R**
8	High-affinity nickel-transport protein (NixA) (3)	G-**H**, **D**-X-**D**-**H**-X(4)-**D**
9	Nickel/cobalt efflux system (RcnA) (16)	**HHHH, EH, HEHD, HDHD, DHHH, EHH**
10	Nickel ABC transporter, periplasmic nickel-binding protein (43)	N-P-**H**-[LVNKER]-Y, N-X(3)-**H**-X-W
11	Putative nickel-responsive regulator (25)	[QKR]-**H**-[EDNH]-[YFH], [STA]-[NQTSV]-[FVLTI]-**H**-[NSIVLMF]-**H**
12	Fused nickel transport protein (NikMN) (6)	**H**-X(6,7)-**E**
13	Hydrogenase nickel incorporation protein (HupN) (11)	D-X-D-**H**-X(4)-D, G-**H**

The table shows the nickel binding patterns observed from the conserved regions of different classes of nickel binding proteins. Letters highlighted in bold indicate the most probable coordinating residues within the respective pattern. The number of sequences for each class of protein considered for pattern analysis is given within brackets. The number of sequences for each class of protein considered for pattern analysis is given within brackets. Only reviewed entries from UniProtKB/Swiss-Prot database were selected for pattern analysis.

Possibility of a single residue chelate was noted in nickel-cobalt-cadmium resistant proteins which showed no conserved histidine or cysteine. Contrarily, the hydrophobic residue alanine and basic residue arginine were highly conserved. Since arginine has the potential to individually coordinate [28] with the metal (PDB ID: 3ONI_A) the residue is considered more optimal for nickel binding in nickel resistant proteins. To ensure this further, the proximal residues of the coordinating arginine at 406^th^ position (Arg406) in the structure (PDB ID 3ONI_A) was noted. Arg406 was bordered by the hydrophilic residues aspartic acid and arginine in the structure. Similarly the sequence alignment of the nickel resistant proteins revealed a hydrophilic environment around arginine with the residues glutamic acid, glutamine, lysine and glycine. Therefore it is projected that arginine could be involved in coordination in nickel resistant proteins. Also in the nickel transport proteins NikQ, the residues aspartic acid and arginine were conserved and therefore have a choice of a single residue chelate. The classes of proteins analysed and their probable nickel binding patterns are listed in Table 4. The analysis of all the nickel binding patterns reveal that nickel coordination is highly selective and specific to a group of proteins and the designing of nickel chelator requires the knowledge of its environment within the proteins. However positional specificity of the chelate residues in the case of cysteine and histidine chelates may provide insight to designing efficient chelate designing.

### Insights derived from coordination analysis

The analysis of nickel binding proteins (sequence) and nickel bound proteins (structure) revealed several nickel binding motifs however, no specificity or selectivity was noted to be associated with nickel binding. Nevertheless, the choice of residue towards nickel coordination was largely attributed to histidine in combination with histidine, aspartic acid and glutamic acid. For histidine the n, n+2, n+4, n+6 spacing was noted to be much preferred. Histidine-aspartic acid chelate followed a regular n, n+2 arrangement and histidine-glutamic acid chelates in n, n+ 3 orders. The presence of an arginine or lysine following a histidine at n+2 or n+3 was also noted to involve in nickel binding. The sequence-structure comparison showed cysteine to equally contribute to nickel coordination. The positional spacing of cysteine was noted to be in the order of n, n+2 and a stretch of 4 cysteine are optimal for chelate designing. Although no specific structural patterns were observed, a random coil is often preferred due to flexibility issues. Therefore presence of a glycine preferably alternating the coordinating residues is believed to provide a flexible architecture of the chelate peptide. In designing chelates, helical chelates are considered a better choice due to their defined geometry in n, n+ 3 orders, for sheets n, n+1 or n+22 can be assigned. Despite the presence of many solvents atoms and heteroatom within the coordination sphere the presence of a single protein donor is mandatory implying the need of a protein chelate. Though most structures show a single residue chelation it cannot be applied for chelate designing without the knowledge of neighboring residues. Also, due to the presence of multiple binding sites it may lead to non-specific binding as a result, a longer chelate is often preferred. From the analysis of several binding motifs, a chelate length of 5–8 donor atoms is predicted to be an ideal length in nickel chelator designing. In addition, the placing of residues and modeling of the peptide into a preferable secondary structural geometry is the vital issue to be implemented to bend the peptide into an effective chelator.

### Conclusion

The present study puts forth important findings from the nickel coordination analysis made on a large dataset of nickel bound proteins and nickel binding proteins. The study showed that histidine plays a major role in nickel coordination either individually or in combination with histidine, aspartic acid, glutamic acid and cysteine. We also report that nickel coordination is purely random with no specific association to the functional fold of protein, or with coordinating residues, whereas the coordinating residues follow a specific order in their position within the sequence. The study also revealed that nickel coordination requires atleast a single protein donor despite the presence or absence of solvents and heteroatoms. This indicates the need for a protein based chelator for nickel coordination. The comparison made between the structures and sequences involved in nickel binding show that nickel coordination is organism specific and selective to a class or function of protein. Based on this several nickel binding signatures have been proposed that may be effective in designing potential chelators targeting several classes of nickel binding proteins and covering wide range of organisms. These findings should provide better understanding of nickel environment and therefore aid in chelation to be implemented for therapeutic, bioremediation or other experimental purposes.

## Material and Methods

### Construction of data set

For the construction of dataset all possible nickel bound proteins were retrieved from Protein Data Bank [31], a repository for 3-dimentional structure of biological macromolecules. To provide uniformity in the analysis, only X-ray crystallographic structures were considered and all NMR and theoretical models were excluded. The initial dataset comprised about 928 crystal structures. The dataset was further was culled at 40% sequence identity using PISCES server [32] to remove redundant structures due to homodimers and identical entries. Other threshold parameters employed included resolution between 0.0–3.0Å and R value of 0.3. The resulting culled dataset was further categorized based on resolution into group A (0–2.0Å), group B (2.1–3.0Å) and group C (3Å and above). This resulted in 186 structures in group A, 135 structures in group B and only 3 structures in group C, therefore group C was ignored for further study. Only a single polypeptide chain from each structure was considered for analysis to avoid repetition of data due to homodomain structures. Further, we also categorized the proteins based on their functional folds obtained from the SCOP and CATH databases [25] to make the analysis more specific towards chelation. All the structures and their coordination geometries were visualized and displayed using PyMol [30].

### Validation and coordination geometry analysis

Further validation of dataset required the analysis of metal coordinating residues. About 321 nickel bound structures from group A and group B were subjected to validation using B-factor and occupancy parameters of nickel coordinating residues. The metal coordinating residues were predicted using ANAMBS [33] with a distance cutoff of 3Å [24] covering the residues and hetero atoms that occur within the first shell [34]. Features like residues, residue position, and atoms of the residues were identified and represented in terms of chelate loop, chelate size, coordination number, donor atoms and non-residue donors. We performed Kruskal Wallis test to find statistical significance in predominant residue and atom that coordinate with nickel (p value < 0.05 was considered statistically significant). In addition the influence of metal ion in stabilizing the secondary structural elements of proteins was estimated as a factor of residue dihedrals that coordinates the metal. The backbone dihedrals were predicted using SwissPDBviewer [29] and represented as Ramachandran plot. Following coordination analysis, the B-factor and occupancy values of the coordinating residues from each of the structures were predicted using Sculptor [35] a multi-scale modeling program for biological macromolecules. Structures having residue B-factors as low as 2.0Å^2^ or lesser and residue occupancies beyond the range 0.5–1.0 were excluded. A correlation graph was plotted between the mean B-factor of the coordinating residues and metal B-factor and the outliers that exceed an average deviation of ≥7.0 were identified and excluded, which resulted in a final dataset comprising 186 structures. These structures were subjected for further analysis.

### Prediction of metal coordination patterns from nickel bound structure

Sequence patterns are defined as a stretch of residues that pose a structural or functional significance in a protein [36]. The main objective of pattern prediction was to design prepositions for a peptide based chelation of nickel ion. Consequently, identifying such patterns provide an insight in the choice of chelate designing. From the coordination data derived with 186 nickel bound structures, we tried to infer unique patterns in nickel coordination. However, in protein structures, residues involved in coordination may primarily be positioned apart but brought closer due to folding. Such long loops exceed the suitable length for pattern writing or chelate designing. To account for this discrepancy, we selected all coordinating patterns that have minimum of two residues separated at distance of ≤10 residue length. For every coordinating residue it’s preceding and succeeding amino acids along with their position within 10 residue length was observed to derive possible coordination patterns. Further we also performed mutual information analysis to validate the co-occurrence derived from the coordination pattern table [26].

### Signature prediction from nickel binding proteins

A signature is a "highly conserved region", a sequence pattern that is found repeatedly in a group of related protein sequences [37]_._ Signatures can be defined for protein family, domains, active site or any other functionally important sites based on the conservation among sequences [36]. All proteins exhibit a structure-function relationship. Since the structures of nickel binding proteins is not been well studied, we made an attempt to predict nickel binding sites of these proteins by comparing the coordination patterns obtained from nickel bound structures. Sequence motifs having high resemblance with the structural patterns were predicted to be more potential in coordinating nickel atom.

### Selection of dataset for multiple sequence alignment

Nickel binding proteins from several archeal and bacterial species were retrieved from UniProtKB/Swiss-Prot database [38]. Only the reviewed entries from UniProtKB/Swiss-Prot database were considered. The sequences were clustered at 90% sequence identity in order to reduce sequence redundancy and the representative sequence from each clusters were selected. The resulting sequences were subjected to conservation analysis through multiple sequence alignment using clustalW server at EBI database [39, 40]. A slow alignment method using a Gonnet matrix with gap open and extension penalties of 10 and 0.1 was used for pairwise alignment. For multiple sequence alignment the matrix remained the same, the gap was open and extension penalties of 10 and 0.2 were used. The minimum gap distance was set to 15, no end-gap values were used and no iterations were specified. The conserved region of multiple sequence alignment that has a residue occurring at a specific position in more than 50% of sequences in the alignment was selected to identify patterns. The number of protein entries selected for multiple sequence alignment is included in Table 4.

### Determination of nickel binding patterns and validation

The nickel binding patterns were predicted from conserved regions within sequences identified through multiple sequence alignment. Segments of sequences devoid of gaps and sharing high sequence identity were considered conserved. However, mutations causing interspecies variation are likely to be more important in defining an organism. Therefore along with sequence identity, regions showing conserved substitutions and semi-conserved substitutions among the group of sequences under investigation were also considered for signature prediction. The patterns were written in the regular PROSITE format and were further validated using scanprosite [41]. Each of the patterns predicted were scanned across UniProtKB/Swiss-Prot and UniProtKB/TrEMBL database [42], a protein repository for curated and annotated sequences. Best patterns were identified as the ones that match maximum number of proteins belonging to that family but show dissimilarity to unrelated proteins. Patterns common for large number of genera were considered favorable. The sequence patterns were further cross-validated with coordination patterns derived from the nickel bound structures. The search was made at low stringency to include variations among functionally distinct structures and sequences. Residue occurrences within the conserved regions showing the identical succeeding and preceding residues at the respective position as observed from the structural motifs were considered the most potential nickel binding site.

## Supporting Information

S1 Table Coordination details of nickel binding from nickel bound proteins. (DOC)

S1 Fig Correlation of metal B-factor and mean B-factor of coordinating residues. The R^2^ value given in the rectangular box indicates the correlation obtained between metal B-factor and residue B-factor. Higher the coorelation value, closer is the relationship between the scores.
(TIF)

S2 Fig Residue preferences in nickel coordination. Figure represents the Statistical significance among nickel coordinating residue based on Kruskal Wallis test [p-value = >0.0001]
(TIF)

S3 Fig Residue preferences in nickel coordination. Figure represents the Statistical significance among nickel coordinating atom based on Kruskal Wallis test [p-value = 0.0001]
(TIF)

S4 Fig Correlation of the average metal-donor distance between high resolution and medium resolution structures. The figure shows the correlation plot for average distance of every atom type involved in nickel coordination. The R^2^ value given in the rectangular box indicates the correlation obtained between metal B-factor and residue B-factor. Higher the coorelation value, closer is the relationship between the scores.
(TIF)

S5 Fig Mutual information analysis on nickel binding site residues. The figure shows the sequence logo for the residues within the coordination sphere. The strength of the letter indicates its predominance at the specific positions. Colors represent the nature of aminoacids where red is acidic aminoacids, blue represents basic aminoacids, green is amide residues and black is hydrophobic amino acids.
(TIF)

S6 Fig Phi-Psi angle distribution of coordinating residues. The figure represents the Ramachandran plot showing the phi (φ) and psi (ψ) angle distribution of chelate residues in a) helix b) sheet and c) turns/coils. Figure (a) shows helices to be well confined compared to sheets and coils.
(TIF)

S7 Fig Superimposed structures of most prominent nickel chelates. The coordinating residues are displayed as sticks and the atom coloring represents carbon (green), nitrogen (blue), oxygen (red) and sulphur (yellow). Nickel atom is shown as sphere and colored in grey. S5A Fig shows the tetra-residue chelates in distorted see-saw geometry. Fig 5b displays the trigonal geometries of tri-residue chelates, amongst which, the HHH chelate (e) shows a well demonstrated coordination. Amongst the bi-residue chelates EE (f) and HH (g), histidine based chelates display a well correlated geometry.
(TIF)
