# Supplementary material for: Ab Initio Coordination Chemistry for Nickel Chelation Motifs
Source: PLoS One. 2015 May 18;10(5):e0126787. doi: 10.1371/journal.pone.0126787 (PMC4435748; doi:10.1371/journal.pone.0126787)
Supplement: S1 Table — (DOC) [file pone.0126787.s001.doc]

**Table S1**

***Ab initio* coordination chemistry for nickel chelation motifs**

R.Jesu Jaya Sudan, J. Lesitha Jeevakumari, *C.Sudandiradoss

Bioinformatics Division, SBST, VIT University, Vellore, India- 632 014

Table S1 Coordination details of nickel binding from nickel bound proteins

| **pdb id^a^** | **Chain^b^** | **posn^c^** | **nspan^d^** | **np^e^** | **nw^f^** | **nn^g^** | **dons^h^** | **met^i^** | **sd1^j^** | **sd2^j^** | **sd3^j^** | **sd4^j^** | **sd5^j^** | **sd6^j^** | **sd7^j^** | **cn^k^** | **res^l^** | **SS^m^** | **Fold/ Architecture^n^** | **Dentate** |
| --- | --- | --- | --- | --- | --- | --- | --- | --- | --- | --- | --- | --- | --- | --- | --- | --- | --- | --- | --- | --- |
| 1EK0 | 1EK0_A | 54 | - | 1 | 4 | 1 | H | NI | -1 | -1 | -1 | -1 | -1 | - | - | 6 | 1.48 | S | P-loop containing nucleoside triphosphate hydrolases | m |
| 1ELR | 1ELR_A | 247 | - | 1 | 3 | 0 | H | NI | -1 | -1 | -1 | - | - | - | - | 4 | 1.9 | H | alpha-alpha superhelix | m |
| 1ELW | 1ELW_A | 101 | - | 1 | 0 | 2 | H | NI | -1 | -1 | - | - | - | - | - | 3 | 1.6 | T | alpha-alpha superhelix | m |
| 1G2A | 1G2A_A | 90 | 46 | 3 | 0 | 1 | CHH | NI | 42 | 4 | -1 | - | - | - | - | 4 | 1.75 | THH | Peptide deformylase | mmm |
| 1H5Q | 1H5Q_A | 262 | - | 1 | 2 | 0 | W | NI | -1 | -1 | - | - | - | - | - | 3 | 1.5 | T | NAD(P)-binding Rossmann-fold domains | m |
| 1H9R | 1H9R_B | 139 | 7 | 3 | 1 | 0 | DDH | NI | 2 | 5 | -1 | - | - | - | - | 4 | 1.9 | STS | OB-fold | mmm |
| 1HBK | 1HBK_A | 49 | - | 1 | 1 | 0 | H | NI | -1 | - | - | - | - | - | - | 2 | 2 | T | Acyl-CoA binding protein-like | m |
| 1HE1 | 1HE1_A | 211 | - | 1 | 3 | 0 | H | NI | -1 | -1 | -1 | - | - | - | - | 4 | 2 | H | Four-helical up-and-down bundle | m |
| 1HYO | 1HYO_B | 499 | - | 1 | 3 | 0 | G | NI | -1 | -1 | -1 | - | - | - | - | 4 | 1.3 | T | SH3-like barrel | b |
| 1IA6 | 1IA6_A | 1 | 343 | 3 | 1 | 0 | AHD | NI | 4 | 339 | -1 | - | - | - | - | 4 | 1.8 | TTT | Alpha/alpha barrel | bmm |
| 1IA7 | 1IA7_A | 1 | 342 | 3 | 1 | 0 | AHD | NI | 3 | 339 | -1 | - | - | - | - | 4 | 2 | TTT | alpha/alpha toroid | bmm |
| 1J5Y | 1J5Y_A | 79 | 67 | 3 | 1 | 0 | HEH | NI | 8 | 59 | -1 | - | - | - | - | 4 | 2.3 | SHS | DNA/RNA-binding 3-helical bundle | mmm |
| 1J6P | 1J6P_A | 55 | 224 | 4 | 2 | 0 | HHHD | NI | 2 | 143 | 79 | -1 | -1 | - | - | 6 | 1.9 | TTST | Alpha-Beta Barrel | mmmm |
| 1JVL | 1JVL_A | 1 | - | 1 | 1 | 0 | A | NI | -1 | - | - | - | - | - | - | 2 | 2 | T | Cupredoxin-like | b |
| 1KIC | 1KIC_A | 44 | 69 | 2 | 0 | 1 | EE | NI | 69 | -1 | - | - | - | - | - | 3 | 1.6 | HH | Nucleoside hydrolase | mm |
| 1KIE | 1KIE_A | 44 | 69 | 2 | 1 | 0 | EE | NI | 69 | -1 | - | - | - | - | - | 3 | 2 | HH | Nucleoside hydrolase | mm |
| 1MN0 | 1MN0_A | 13 | - | 1 | 1 | 0 | D | NI | -1 | - | - | - | - | - | - | 2 | 1.9 | S | Supersandwich | m |
| 1OAO | 1OAO_C | 595 | 2 | 3 | 0 | 1 | CGC | NI | 1 | 1 | -1 | - | - | - | - | 4 | 1.9 | TTT | Prismane protein-like | mmb |
| 1OEJ | 1OEJ_A | 144 | 11 | 3 | 1 | 0 | HHH | NI | 9 | 2 | -1 | - | - | - | - | 4 | 1.81 | TTS | Lipocalins | mmm |
| 1OPM | 1OPM_A | 235 | - | 1 | 1 | 2 | H | NI | -1 | -1 | -1 | - | - | - | - | 4 | 2.1 | S | Nucleoplasmin-like/VP (viral coat and capsid proteins) | m |
| 1Q5Y | 1Q5Y_A | 87 | 8 | 3 | 0 | 0 | HHC | NI | 2 | 6 | - | - | - | - | - | 3 | 1.4 | SSS | Ferredoxin-like | mmm |
| 1QWZ | 1QWZ_A | 17 | 2 | 2 | 0 | 0 | HH | NI | 2 | - | - | - | - | - | - | 2 | 1.75 | TT | Sortase | mm |
| 1QXJ | 1QXJ_A | 88 | 48 | 4 | 2 | 0 | HHEH | NI | 2 | 7 | 39 | -1 | -1 | - | - | 6 | 1.15 | TTSS | Double-stranded beta-helix | mmm |
| 1QY7 | 1QY7_A | 1 | 109 | 3 | 2 | 0 | MVD | NI | 95 | 14 | -1 | -1 | - | - | - | 5 | 2 | SSS | Ferredoxin-like | mmm |
| 1RXQ | 1RXQ_A | 67 | 97 | 3 | 3 | 0 | HHH | NI | 93 | 4 | -1 | -1 | -1 | - | - | 6 | 1.7 | HHH | Up-down Bundle | mmm |
| 1S3Z | 1S3Z_A | 0 | 2 | 2 | 0 | 0 | HD | NI | 2 | - | - | - | - | - | - | 2 | 2 | TS | Acyl-CoA N-acyltransferases (Nat) | mm |
| 1SCR | 1SCR_A | 8 | 16 | 4 | 2 | 0 | EDDH | NI | 2 | 9 | 5 | -1 | -1 | - | - | 6 | 2 | SSTS | Concanavalin A-like lectins/glucanases | mmmm |
| 1SDW | 1SDW_A | 235 | - | 1 | 2 | 1 | H | NI | -1 | -1 | -1 | - | - | - | - | 4 | 1.85 | S | Nucleoplasmin-like/VP (viral coat and capsid proteins) | m |
| 1SLW | 1SLW_A | 86 | - | 1 | 0 | 0 | H | NI | - | - | - | - | - | - | - | 1 | 2 | T | Ecotin, trypsin inhibitor | m |
| 1T6U | 1T6U_A | 2 | 4 | 2 | 0 | 0 | CC | NI | 4 | - | - | - | - | - | - | 2 | 1.3 | TT | Up-down Bundle | bm |
| 1UMH | 1UMH_A | 114 | 1 | 2 | 0 | 0 | GS | NI | 1 | - | - | - | - | - | - | 2 | 2 | TT | Galactose-binding domain-like | mb |
| 1VJR | 1VJR_A | 0 | - | 1 | 0 | 2 | H | NI | -1 | -1 | - | - | - | - | - | 3 | 2.4 | T | HAD-like | m |
| 1VKL | 1VKL_A | 287 | 4 | 3 | 0 | 0 | DDD | NI | 2 | 2 | - | - | - | - | - | 3 | 2.7 | TTT | Phosphoglucomutase, first 3 domains | mmm |
| 1VR3 | 1VR3_A | 88 | 45 | 4 | 0 | 1 | HHEH | NI | 2 | 4 | 39 | -1 | - | - | - | 5 | 2.06 | STSS | Double-stranded beta-helix | mmmm |
| 1W9H | 1W9H_A | 73 | - | 1 | 1 | 0 | D | NI | -1 | - | - | - | - | - | - | 2 | 1.95 | H | Ribonuclease H-like motif | m |
| 1WU4 | 1WU4_A | 27 | 3 | 2 | 0 | 0 | EE | NI | 3 | - | - | - | - | - | - | 2 | 1.35 | HH | alpha/alpha toroid | bm |
| 1XI3 | 1XI3_B | 26 | 3 | 2 | 0 | 0 | EE | NI | 3 | - | - | - | - | - | - | 2 | 1.7 | HH | TIM beta/alpha-barrel | bb |
| 1XMK | 1XMK_A | 288 | 2 | 3 | 0 | 0 | GSH | NI | 1 | 1 | - | - | - | - | - | 3 | 0.97 | TTT | DNA/RNA-binding 3-helical bundle | mmb |
| 1XU2 | 1XU2_A | 106 | - | 1 | 0 | 0 | H | NI | - | - | - | - | - | - | - | 1 | 2.35 | T | TNF-like | m |
| 1Y1O | 1Y1O_A | 151 | 24 | 3 | 1 | 0 | HEH | NI | 20 | 4 | -1 | - | - | - | - | 4 | 2.2 | HHH | Restriction endonuclease-like | mmm |
| 1YSJ | 1YSJ_A | 98 | 60 | 3 | 2 | 0 | CHH | NI | 2 | 58 | -1 | -1 | - | - | - | 5 | 2.4 | THS | Phosphorylase/hydrolase-like | mmm |
| 1ZLE | 1ZLE_A | 83 | 2 | 2 | 0 | 0 | DD | NI | 2 | - | - | - | - | - | - | 2 | 1.9 | SH | NA | mm |
| 1ZNV | 1ZNV_B | 544 | 29 | 3 | 0 | 1 | HHH | NI | 25 | 4 | -1 | - | - | - | - | 4 | 2 | SHH | Acyl carrier protein-like | mmm |
| 1ZTC | 1ZTC_A | 73 | 126 | 4 | 2 | 0 | DHDH | NI | 1 | 88 | 37 | -1 | -1 | - | - | 6 | 2.1 | HHT | Metallo-hydrolase/oxidoreductase | mmm |
| 260L | 260L_A | 21 | 121 | 2 | 4 | 0 | HH | NI | 121 | -1 | -1 | -1 | -1 | - | - | 6 | 1.8 | TT | Lysozyme-like | mm |
| 2AFB | 2AFB_A | 67 | - | 1 | 2 | 0 | H | NI | -1 | -1 | - | - | - | - | - | 3 | 2.05 | H | Ribokinase-like | m |
| 2BDG | 2BDG_A | 25 | 52 | 2 | 4 | 0 | HE | NI | 52 | -1 | -1 | -1 | -1 | - | - | 6 | 1.95 | TT | Beta Barrel | mm |
| 2BJ7 | 2BJ7_A | 89 | 8 | 3 | 0 | 0 | HHC | NI | 2 | 6 | - | - | - | - | - | 3 | 2.1 | SSS | Ribbon-helix-helix | mmm |
| 2BMO | 2BMO_B | 14 | 146 | 2 | 3 | 0 | HE | NI | 146 | -1 | -1 | -1 | - | - | - | 5 | 1.2 | HS | ISP domain | mm |
| 2C0N | 2C0N_A | 198 | 2 | 2 | 0 | 0 | HH | NI | 2 | - | - | - | - | - | - | 2 | 1.86 | TT | NA | mm |
| 2C21 | 2C21_B | 380 | 51 | 2 | 1 | 0 | HH | NI | 51 | -1 | - | - | - | - | - | 2 | 2 | SS | Glyoxalase/Bleomycin resistance protein/Dihydroxybiphenyl dioxygenase | mm |
| 2C9W | 2C9W_A | 111 | - | 1 | 0 | 0 | C | NI | - | - | - | - | - | - | - | 1 | 1.9 | T | beta-Grasp (ubiquitin-like roll) | m |
| 2D58 | 2D58_A | 66 | - | 1 | 5 | 0 | D | NI | -1 | -1 | -1 | -1 | -1 | - | - | 6 | 1.9 | T | NA | m |
| 2DRS | 2DRS_A | 27 | 3 | 2 | 0 | 0 | EE | NI | 3 | - | - | - | - | - | - | 2 | 2.1 | HH | NA | mm |
| 2EAQ | 2EAQ_A | 1116 | - | 1 | 2 | 0 | H | NI | -1 | -1 | - | - | - | - | - | 3 | 1.46 | S | NA | m |
| 2F22 | 2F22_A | 42 | 76 | 3 | 3 | 0 | HHH | NI | 72 | 4 | -1 | -1 | -1 | - | - | 6 | 1.42 | HHH | Up-down Bundle/DinB/YfiT-like putative metalloenzymes | mmm |
| 2FJ1 | 2FJ1_A | 100 | - | 1 | 3 | 1 | H | NI | -1 | -1 | -1 | -1 | - | - | - | 5 | 2.2 | H | DNA/RNA-binding 3-helical bundle | m |
| 2GLZ | 2GLZ_A | 15 | 40 | 4 | 0 | 1 | HHCC | NI | 2 | 2 | 36 | -1 | - | - | - | 5 | 1.45 | HTTT | FwdE/GAPDH domain-like | mmmm |
| 2GU3 | 2GU3_A | 53 | - | 1 | 3 | 0 | H | NI | -1 | -1 | -1 | - | - | - | - | 4 | 1.74 | S | Cystatin-like | m |
| 2HJE | 2HJE_A | 209 | 62 | 2 | 3 | 0 | DD | NI | 62 | -1 | -1 | -1 | - | - | - | 5 | 1.7 | TT | Profilin-like | mm |
| 2HKV | 2HKV_A | 48 | 79 | 3 | 2 | 1 | HHH | NI | 75 | 4 | -1 | -1 | -1 | - | - | 6 | 1.7 | HHH | Up-down Bundle | mmm |
| 2I2O | 2I2O_A | 119 | - | 1 | 0 | 0 | N | NI | - | - | - | - | - | - | - | 1 | 1.92 | T | Alpha Horseshoe | m |
| 2ISY | 2ISY_A | 79 | 19 | 3 | 2 | 1 | HEH | NI | 4 | 15 | -1 | -1 | -1 | - | - | 6 | 1.96 | HHH | DNA/RNA-binding 3-helical bundle | mbm |
| 2IWB | 2IWB_A | 450 | - | 1 | 0 | 0 | H | NI | - | - | - | - | - | - | - | 1 | 1.8 | T | 3-Layer(aba) Sandwich | m |
| 2IXL | 2IXL_A | 52 | - | 1 | 2 | 0 | E | NI | -1 | -1 | - | - | - | - | - | 3 | 1.6 | T | Double-stranded beta-helix | m |
| 2NOO | 2NOO_A | 56 | 386 | 2 | 4 | 0 | HH | NI | 386 | -1 | -1 | -1 | -1 | - | - | 6 | 1.65 | ST | NA | mm |
| 2NQC | 2NQC_A | 2532 | 7 | 2 | 0 | 2 | CH | NI | 7 | -1 | -1 | - | - | - | - | 4 | 2.05 | SS | Immunoglobulin-like beta-sandwich | mm |
| 2O8Q | 2O8Q_A | 58 | 42 | 3 | 4 | 0 | HHH | NI | 2 | 40 | -1 | -1 | -1 | -1 | - | 7 | 1.55 | SS | Sandwich | mmm |
| 2OQ6 | 2OQ6_A | 188 | 88 | 3 | 1 | 1 | HEH | NI | 2 | 86 | -1 | -1 | - | - | - | 5 | 2 | TTS | NA | mbm |
| 2OS0 | 2OS0_A | 114 | 47 | 3 | 1 | 0 | CHH | NI | 43 | 4 | -1 | - | - | - | - | 4 | 1.3 | THH | Alpha-Beta Complex | mmm |
| 2OU6 | 2OU6_A | 76 | 96 | 3 | 3 | 0 | HDH | NI | 92 | 4 | -1 | -1 | -1 | - | - | 6 | 1.8 | HHH | DinB/YfiT-like putative metalloenzymes | mmm |
| 2P0U | 2P0U_B | 3 | 5 | 3 | 0 | 0 | HHH | NI | 3 | 2 | - | - | - | - | - | 3 | 1.9 | SSS | NA | mmm |
| 2POS | 2POS_A | 1 | - | 1 | 0 | 1 | W | NI | -1 | - | - | - | - | - | - | 2 | 1.6 | T | NA | b |
| 2PW8 | 2PW8_L | 1 | 8 | 2 | 3 | 0 | DK | NI | 8 | -1 | -1 | -1 | - | - | - | 5 | 1.84 | TT | Beta Barrel | mm |
| 2Q4S | 2Q4S_A | 86 | 54 | 3 | 3 | 0 | HHH | NI | 2 | 52 | -1 | -1 | -1 | - | - | 6 | 1.75 | TTS | Double-stranded beta-helix | mmm |
| 2QE9 | 2QE9_A | 44 | 84 | 3 | 0 | 1 | HHH | NI | 80 | 4 | -1 | - | - | - | - | 4 | 1.9 | HHH | Up-down Bundle | mmm |
| 2QNK | 2QNK_A | 47 | 44 | 3 | 2 | 0 | HEH | NI | 6 | 38 | -1 | -1 | - | - | - | 5 | 1.6 | SSS | NA | mbm |
| 2QQA | 2QQA_A | 79 | 19 | 3 | 2 | 1 | HEH | NI | 4 | 15 | -1 | -1 | -1 | - | - | 6 | 2.1 | HHH | NA | mmm |
| 2R9Z | 2R9Z_A | 120 | - | 1 | 3 | 0 | H | NI | -1 | -1 | -1 | - | - | - | - | 4 | 2.1 | T | 3-Layer(bba) Sandwich | m |
| 2RD9 | 2RD9_A | 48 | 98 | 3 | 1 | 1 | HHH | NI | 94 | 4 | -1 | -1 | - | - | - | 5 | 2.3 | HHH | Up-down Bundle | mmm |
| 2RJ2 | 2RJ2_A | 113 | 2 | 3 | 0 | 0 | GSH | NI | 1 | 1 | - | - | - | - | - | 3 | 1.7 | TTT | NA | mmb |
| 2UUI | 2UUI_A | 1 | - | 1 | 0 | 0 | H | NI | - | - | - | - | - | - | - | 1 | 2 | T | MAPEG domain-like | m |
| 2VTC | 2VTC_A | 1 | 176 | 3 | 2 | 0 | HHY | NI | 89 | 87 | -1 | -1 | - | - | - | 5 | 1.6 | TTS | NA | bmm |
| 2VUW | 2VUW_A | 477 | 86 | 2 | 3 | 0 | HH | NI | 86 | -1 | -1 | -1 | - | - | - | 5 | 1.8 | HS | NA | mm |
| 2W37 | 2W37_A | 79 | - | 1 | 1 | 0 | H | NI | -1 | - | - | - | - | - | - | 2 | 2.1 | S | 3-Layer(aba) Sandwich | m |
| 2W95 | 2W95_A | 16 | - | 1 | 0 | 0 | H | NI | - | - | - | - | - | - | - | 1 | 1.75 | S | NA | m |
| 2WCR | 2WCR_A | 157 | - | 1 | 0 | 1 | H | NI | -1 | - | - | - | - | - | - | 2 | 1.7 | H | NA | m |
| 2WNY | 2WNY_A | 45 | - | 1 | 0 | 0 | H | NI | - | - | - | - | - | - | - | 1 | 1.95 | T | NA | m |
| 2WPN | 2WPN_B | 75 | 417 | 3 | 0 | 1 | CCC | NI | 3 | 414 | -1 | - | - | - | - | 4 | 2.04 | TTH | 3-Layer(aba) Sandwich | mmm |
| 2WQM | 2WQM_A | 209 | 38 | 2 | 0 | 0 | HC | NI | 38 | - | - | - | - | - | - | 2 | 2.1 | TH | Orthogonal Bundle | mm |
| 2X27 | 2X27_X | 173 | 25 | 3 | 1 | 0 | DDD | NI | 2 | 23 | -1 | - | - | - | - | 4 | 2.4 | SSS | Beta Barrel | mmm |
| 2XAU | 2XAU_A | 479 | - | 1 | 1 | 0 | H | NI | -1 | - | - | - | - | - | - | 2 | 1.9 | T | NA | m |
| 2XDV | 2XDV_A | 179 | 61 | 3 | 1 | 1 | HDH | NI | 2 | 59 | -1 | -1 | - | - | - | 5 | 2.57 | STS | NA | mmm |
| 2XIO | 2XIO_A | 252 | - | 1 | 0 | 0 | H | NI | - | - | - | - | - | - | - | 1 | 1.19 | T | NA | m |
| 2XVL | 2XVL_A | 882 | - | 1 | 0 | 5 | H | NI | -1 | -1 | -1 | -1 | -1 | - | - | 6 | 2.3 | T | NA | m |
| 2Y1H | 2Y1H_A | 231 | - | 1 | 1 | 0 | E | NI | -1 | - | - | - | - | - | - | 2 | 2.5 | T | NA | m |
| 2Y39 | 2Y39_A | 42 | 81 | 5 | 0 | 0 | HHEHM | NI | 4 | 17 | 56 | 4 | - | - | - | 5 | 1.41 | HHHHH | NA | mmbmm |
| 2YG9 | 2YG9_A | 35 | - | 1 | 5 | 0 | H | NI | -1 | -1 | -1 | -1 | -1 | - | - | 6 | 1.95 | H | NA | m |
| 2ZPL | 2ZPL_A | 175 | - | 1 | 3 | 0 | D | NI | -1 | -1 | -1 | - | - | - | - | 4 | 1.7 | T | NA | m |
| 3AVR | 3AVR_A | 1146 | 80 | 3 | 1 | 1 | HEH | NI | 2 | 78 | -1 | -1 | - | - | - | 5 | 1.8 | TTS | NA | mmm |
| 3B2Y | 3B2Y_A | 75 | 94 | 3 | 2 | 0 | HEH | NI | 3 | 91 | -1 | -1 | - | - | - | 5 | 1.74 | TTS | DNA/RNA-binding 3-helical bundle | mbm |
| 3BIX | 3BIX_A | 640 | 2 | 2 | 0 | 0 | HH | NI | 2 | - | - | - | - | - | - | 2 | 1.8 | ST | 3-Layer(aba) Sandwich | mm |
| 3BJD | 3BJD_A | 181 | 93 | 3 | 3 | 0 | EHH | NI | 10 | 83 | -1 | -1 | -1 | - | - | 6 | 1.85 | HHH | Orthogonal Bundle | mmm |
| 3C6C | 3C6C_A | 47 | 203 | 3 | 3 | 0 | HHE | NI | 2 | 201 | -1 | -1 | -1 | - | - | 6 | 0.15 | STT | Alpha-Beta Barrel | mmm |
| 3C7J | 3C7J_A | 144 | 70 | 4 | 2 | 0 | NHHH | NI | 4 | 44 | 22 | -1 | -1 | - | - | 6 | 2.1 | HHHH | Orthogonal Bundle | mmmm |
| 3CGM | 3CGM_A | 145 | 12 | 5 | 0 | 0 | HHHHH | NI | 2 | 2 | 6 | 2 | - | - | - | 5 | 2.41 | HTTTT | NA | mmmmm |
| 3CU2 | 3CU2_A | 43 | 149 | 4 | 2 | 0 | HDHD | NI | 2 | 29 | 118 | -1 | -1 | - | - | 6 | 1.91 | SSSS | Alpha-Beta Barrel | mmmm |
| 3D34 | 3D34_A | 175 | - | 1 | 4 | 0 | N | NI | -1 | -1 | -1 | -1 | - | - | - | 5 | 1.8 | T | NA | m |
| 3DKQ | 3DKQ_A | 96 | 61 | 3 | 0 | 2 | HDH | NI | 2 | 59 | -1 | -1 | - | - | - | 5 | 2.26 | TSS | NA | mmm |
| 3DSE | 3DSE_A | 269 | 3 | 2 | 3 | 0 | HK | NI | 3 | -1 | -1 | -1 | - | - | - | 5 | 1.5 | TH | NA | mm |
| 3E3U | 3E3U_A | 106 | 46 | 3 | 0 | 1 | CHH | NI | 42 | 4 | -1 | - | - | - | - | 4 | 1.56 | THH | Alpha-Beta Complex | mmm |
| 3ESK | 3ESK_A | 321 | 4 | 2 | 2 | 0 | HK | NI | 4 | -1 | -1 | - | - | - | - | 4 | 2.05 | HH | Alpha Horseshoe | mm |
| 3FMS | 3FMS_A | 134 | 62 | 3 | 0 | 1 | HHH | NI | 40 | 22 | -1 | - | - | - | - | 4 | 2.2 | HHH | NA | mmm |
| 3G4X | 3G4X_A | 1 | 5 | 3 | 0 | 0 | HCC | NI | 1 | 4 | - | - | - | - | - | 3 | 2.01 | TTT | Up-down Bundle | bbm |
| 3GOR | 3GOR_A | 47 | 84 | 3 | 0 | 0 | HHH | NI | 80 | 4 | - | - | - | - | - | 3 | 2.51 | HHH | NA | mmm |
| 3GRF | 3GRF | 71 | - | 1 | 0 | 0 | H | NI | - | - | - | - | - | - | - | 1 | 2 | S | 3-Layer(aba) Sandwich | m |
| 3H0N | 3H0N_A | 112 | 2 | 2 | 1 | 0 | HH | NI | 2 | -1 | - | - | - | - | - | 3 | 1.45 | ST | Orthogonal Bundle | mm |
| 3HPX | 3HPX_A | 81 | 240 | 4 | 2 | 0 | DHHN | NI | 204 | 2 | 34 | -1 | -1 | - | - | 6 | 2.03 | HSTT | Alpha-Beta Barrel | mmmm |
| 3HT1 | 3HT1_A | 53 | 42 | 4 | 2 | 0 | HHHH | NI | 2 | 4 | 36 | -1 | -1 | - | - | 6 | 1.2 | STST | NA | mmmm |
| 3HTN | 3HTN_A | 131 | 2 | 2 | 0 | 0 | HH | NI | 2 | - | - | - | - | - | - | 2 | 1.5 | SS | NA | mm |
| 3I04 | 3I04_M | 595 | 2 | 3 | 0 | 0 | CGC | NI | 1 | 1 | - | - | - | - | - | 3 | 2.15 | TTT | Up-down Bundle | mmb |
| 3IAR | 3IAR_A | 15 | 280 | 4 | 0 | 1 | HHHD | NI | 2 | 197 | 81 | -1 | - | - | - | 5 | 1.52 | TTST | Alpha-Beta Barrel | mmmm |
| 3IHT | 3IHT_A | 151 | - | 1 | 1 | 0 | E | NI | -1 | - | - | - | - | - | - | 2 | 1.8 | T | NA | m |
| 3II2 | 3II2_A | 77 | 2 | 2 | 0 | 0 | HD | NI | 2 | - | - | - | - | - | - | 2 | 2 | SS | NA | mm |
| 3III | 3III_A | 15 | - | 1 | 4 | 0 | H | NI | -1 | -1 | -1 | -1 | - | - | - | 5 | 1.95 | T | NA | m |
| 3IMP | 3IMP_G | 176 | - | 1 | 0 | 1 | H | NI | -1 | - | - | - | - | - | - | 2 | 2.5 | T | NA | m |
| 3IWF | 3IWF_A | 31 | 69 | 2 | 0 | 0 | HH | NI | 69 | - | - | - | - | - | - | 2 | 1.4 | HT | NA | mm |
| 3K2O | 3K2O_A | 187 | 86 | 3 | 3 | 0 | HDH | NI | 2 | 84 | -1 | -1 | -1 | - | - | 6 | 1.75 | TTS | NA | mmm |
| 3KBN | 3KBN_A | 217 | 3 | 2 | 1 | 1 | EH | NI | 3 | -1 | -1 | - | - | - | - | 4 | 1.53 | TH | Alpha-Beta Barrel | mm |
| 3L6T | 3L6T_A | 149 | 13 | 3 | 0 | 1 | HHH | NI | 11 | 2 | -1 | - | - | - | - | 4 | 1.93 | SSS | NA | mmm |
| 3LAG | 3LAG_A | 32 | 44 | 3 | 1 | 1 | HHH | NI | 2 | 42 | -1 | -1 | - | - | - | 5 | 1.15 | SSS | NA | mmm |
| 3LE0 | 3LE0_A | 80 | - | 1 | 5 | 0 | H | NI | -1 | -1 | -1 | -1 | -1 | - | - | 6 | 1.91 | T | NA | m |
| 3LEG | 3LEG_A | 80 | - | 1 | 5 | 0 | H | NI | -1 | -1 | -1 | -1 | -1 | - | - | 6 | 2.01 | T | NA | m |
| 3LHO | 3LHO_A | 40 | 194 | 3 | 3 | 0 | HHE | NI | 124 | 70 | -1 | -1 | -1 | - | - | 6 | 1.8 | SSS | NA | mmm |
| 3LMW | 3LMW_A | 178 | 320 | 3 | 1 | 0 | DHH | NI | 318 | 2 | -1 | - | - | - | - | 4 | 2.6 | STT | NA | mmm |
| 3LOP | 3LOP_A | 344 | 4 | 2 | 3 | 0 | HE | NI | 4 | -1 | -1 | -1 | - | - | - | 5 | 1.55 | HT | NA | mb |
| 3LXY | 3LXY_A | 167 | 100 | 2 | 3 | 0 | HH | NI | 100 | -1 | -1 | -1 | - | - | - | 5 | 1.7 | TT | 3-Layer(aba) Sandwich | mm |
| 3MS5 | 3MS5_A | 202 | 145 | 3 | 0 | 1 | HDH | NI | 2 | 143 | -1 | - | - | - | - | 4 | 1.82 | TTS | NA | mmm |
| 3N0Q | 3N0Q_A | 201 | 155 | 3 | 0 | 1 | HHD | NI | 5 | 150 | -1 | - | - | - | - | 4 | 1.8 | HTH | NA | mmm |
| 3N0W | 3N0W_A | 344 | - | 1 | 2 | 0 | H | NI | -1 | -1 | - | - | - | - | - | 3 | 1.88 | T | NA | m |
| 3NE7 | 3NE7_A | 100 | - | 1 | 0 | 1 | H | NI | -1 | - | - | - | - | - | - | 2 | 2.3 | T | 3-Layer(aba) Sandwich | m |
| 3NF3 | 3NF3_A | 269 | 3 | 2 | 5 | 0 | HK | NI | 3 | -1 | -1 | -1 | -1 | -1 | - | 7 | 2.4 | TH | NA | mm |
| 3NO4 | 3NO4_A | 31 | 141 | 3 | 3 | 0 | HDE | NI | 9 | 132 | -1 | -1 | -1 | - | - | 6 | 2 | THH | NA | mmm |
| 3NV1 | 3NV1_A | 320 | - | 1 | 1 | 0 | H | NI | -1 | - | - | - | - | - | - | 2 | 1.5 | S | NA | m |
| 3O01 | 3O01_A | 36 | 1 | 2 | 0 | 0 | GH | NI | 1 | - | - | - | - | - | - | 2 | 1.9 | TT | NA | mb |
| 3ONI | 3ONI_A | 406 | - | 1 | 1 | 0 | R | NI | -1 | - | - | - | - | - | - | 2 | 1.61 | T | NA | m |
| 3PHT | 3PHT_A | 99 | 8 | 3 | 0 | 0 | HHC | NI | 2 | 6 | - | - | - | - | - | 3 | 2.04 | SSS | NA | mmm |
| 3PKE | 3PKE_A | 142 | 127 | 4 | 0 | 0 | DHEE | NI | 63 | 33 | 31 | - | - | - | - | 4 | 1.6 | SSTS | Alpha-Beta Complex | mmmm |
| 3PUA | 3PUA_A | 249 | 72 | 3 | 1 | 1 | HDY | NI | 2 | 70 | -1 | -1 | - | - | - | 5 | 1.89 | TTS | NA | mmm |
| 3PUS | 3PUS_A | 249 | 72 | 3 | 1 | 1 | HDY | NI | 2 | 70 | -1 | -1 | - | - | - | 5 | 2.08 | TTS | NA | mmm |
| 3QC3 | 3QC3_A | 35 | 140 | 4 | 1 | 3 | HDHD | NI | 2 | 33 | 105 | -1 | -1 | -1 | -1 | 8 | 2.2 | SSSS | Alpha-Beta Barrel | mmmm |
| 3QD1 | 3QD1_X | 257 | - | 1 | 3 | 0 | H | NI | -1 | -1 | -1 | - | - | - | - | 4 | 1.9 | S | NA | m |
| 3QVQ | 3QVQ_A | 138 | - | 1 | 2 | 0 | H | NI | -1 | -1 | - | - | - | - | - | 3 | 1.6 | T | NA | m |
| 3RF7 | 3RF7_A | 194 | 78 | 4 | 0 | 2 | DHHH | NI | 4 | 60 | 14 | -1 | -1 | - | - | 6 | 2.12 | HHHH | NA | mmmm |
| 3RQT | 3RQT_A | 471 | 1 | 2 | 0 | 0 | HH | NI | 1 | - | - | - | - | - | - | 2 | 1.5 | TT | NA | tt |
| 3RVA | 3RVA_A | 2 | 2 | 3 | 0 | 0 | SQH | NI | 1 | 1 | - | - | - | - | - | 3 | 1.8 | TTH | NA | mmb |
| 3S0K | 3S0K_A | 7 | 22 | 3 | 0 | 0 | HEH | NI | 18 | 4 | - | - | - | - | - | 3 | 1.4 | THT | NA | mbm |
| 3S42 | 3S42_B | 134 | - | 1 | 0 | 1 | H | NI | -1 | - | - | - | - | - | - | 2 | 1.45 | T | Alpha-Beta Barrel | m |
| 3SEX | 3SEX_A | 40 | - | 1 | 0 | 0 | H | NI | - | - | - | - | - | - | - | 1 | 1.95 | T | NA | m |
| 3SKD | 3SKD_A | 24 | 181 | 4 | 2 | 0 | HHDD | NI | 45 | 1 | 135 | -1 | -1 | - | - | 6 | 2 | HTTH | NA | mmmm |
| 3T5N | 3T5N_A | 204 | 22 | 2 | 3 | 0 | DH | NI | 22 | -1 | -1 | -1 | - | - | - | 5 | 1.96 | HT | NA | mm |
| 3T9W | 3T9W_A | 193 | 1 | 2 | 4 | 0 | HH | NI | 1 | -1 | -1 | -1 | -1 | - | - | 6 | 1.5 | TT | NA | mm |
| 3TA2 | 3TA2_B | 15 | - | 1 | 0 | 0 | C | NI | - | - | - | - | - | - | - | 1 | 1.9 | H | NA | m |
| 3TJ8 | 3TJ8_B | 102 | 50 | 2 | 1 | 0 | HH | NI | 50 | -1 | - | - | - | - | - | 3 | 1.59 | TT | NA | mm |
| 3TNG | 3TNG_A | 157 | - | 1 | 0 | 0 | H | NI | - | - | - | - | - | - | - | 1 | 2.16 | H | NA | m |
| 3TOY | 3TOY_A | 64 | - | 1 | 2 | 0 | H | NI | -1 | -1 | - | - | - | - | - | 3 | 1.8 | H | NA | m |
| 3TSN | 3TSN_A | 177 | 124 | 2 | 0 | 1 | HH | NI | 124 | -1 | - | - | - | - | - | 3 | 2.63 | TT | NA | mm |
| 3U8V | 3U8V_A | 1 | 6 | 2 | 0 | 0 | SH | NI | 6 | - | - | - | - | - | - | 2 | 1.9 | TH | NA | bm |
| 3UBP | 3UBP_C | 249 | 26 | 2 | 0 | 1 | HH | NI | 26 | -1 | - | - | - | - | - | 3 | 2 | SS | beta-clip | mm |
| 3UGV | 3UGV_A | 244 | - | 1 | 0 | 0 | H | NI | - | - | - | - | - | - | - | 1 | 2.3 | H | NA | m |
| 3USC | 3USC_L | 76 | 503 | 4 | 1 | 1 | CCCC | NI | 3 | 497 | 3 | -1 | -1 | - | - | 6 | 2 | TTTH | NA | mmmm |
| 3VJ9 | 3VJ9_A | 343 | 4 | 2 | 3 | 0 | EH | NI | 4 | -1 | -1 | -1 | - | - | - | 5 | 1.52 | HT | NA | mm |
| 3ZUC | 3ZUC_A | 1 | 3 | 3 | 0 | 0 | GSH | NI | 1 | 1 | - | - | - | - | - | 3 | 1 | TTT | NA | mmm |
| 4A8P | 4A8P_A | 69 | - | 1 | 3 | 0 | H | NI | -1 | -1 | -1 | - | - | - | - | 4 | 2 | S | NA | m |
| 4A8T | 4A8T_A | 69 | - | 1 | 1 | 0 | H | NI | -1 | - | - | - | - | - | - | 2 | 1.59 | S | NA | m |
| 4AUU | 4AUU_A | 45 | 2 | 2 | 4 | 0 | HD | NI | 2 | -1 | -1 | -1 | -1 | - | - | 6 | 1.6 | ST | NA | mm |
| 4AUY | 4AUY_A | 45 | 2 | 2 | 4 | 0 | HD | NI | 2 | -1 | -1 | -1 | -1 | - | - | 6 | 2.1 | ST | NA | mm |
| 4B29 | 4B29_A | 42 | - | 1 | 2 | 0 | H | NI | -1 | -1 | - | - | - | - | - | 3 | 1.72 | T | NA | m |
| 4DIQ | 4DIQ_A | 340 | 65 | 3 | 1 | 1 | HDH | NI | 2 | 63 | -1 | -1 | - | - | - | 5 | 2.4 | TTS | NA | mmm |
| 4DUN | 4DUN_A | 71 | - | 1 | 0 | 1 | H | NI | -1 | - | - | - | - | - | - | 2 | 1.76 | H | NA | m |
| 4E2G | 4E2G_B | 54 | 40 | 4 | 0 | 0 | HHQH | NI | 2 | 4 | 34 | - | - | - | - | 4 | 1.86 | TTSS | NA | mmmm |
| 4EZ4 | 4EZ4_A | 1390 | 80 | 3 | 0 | 1 | HEH | NI | 2 | 78 | -1 | - | - | - | - | 4 | 2.99 | TTS | NA | mmm |
| 4F9D | 4F9D_A | 115 | 74 | 3 | 1 | 1 | DHH | NI | 69 | 5 | -1 | -1 | - | - | - | 5 | 1.9 | SST | NA | mmm |
| 4GSV | 4GSV_A | 101 | 131 | 3 | 0 | 1 | HDD | NI | 23 | 108 | -1 | - | - | - | - | 4 | 1.48 | TST | NA | mmm |
| 4H2H | 4H2H_A | 235 | - | 1 | 0 | 0 | H | NI | - | - | - | - | - | - | - | 1 | 1.7 | T | NA | m |
| 4UBP | 4UBP_C | 249 | 26 | 2 | 0 | 1 | HH | NI | 26 | -1 | - | - | - | - | - | 3 | 1.55 | SS | TIM beta/alpha-barrel | mm |

^a^ protein ID as given in protein data bank

^b^ Protein ID as given in Protein data bank followed by the chain ID

^c^ position of the first residue in the chelate loop

^d^ represents the length span of the chelate loop

^e^ represents the number of donor atoms

^f^ represents the number of water molecule

^g^ represents the number of non protein donors

^h^ indicate the donor atoms specified as single letter codes

^i^ metal ion,

^j^ positioning of the residues from the first residue of the chelate

^k^ coordination number,

^l^ resolution

^m^ secondary structural elements

^n^ Structural classification according to CATH and SCOP database

^o^ Type of atom coordination. (‘m’ monodentate coordination, ‘b’ bidentate coordination, ‘t’ tridentate coordination)
