# Supplementary figures and images for: Ab Initio Coordination Chemistry for Nickel Chelation Motifs
Source: PLoS One. 2015 May 18;10(5):e0126787. doi: 10.1371/journal.pone.0126787 (PMC4435748; doi:10.1371/journal.pone.0126787)

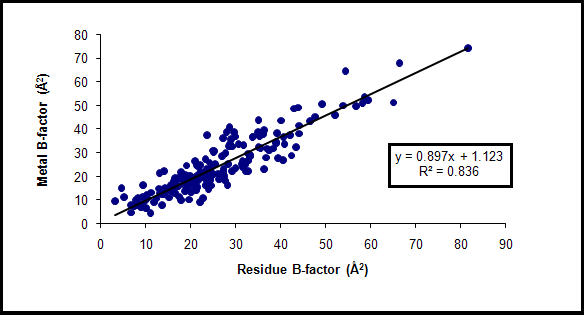

Supplement: S1 Fig — The R2 value given in the rectangular box indicates the correlation obtained between metal B-factor and residue B-factor. Higher the coorelation value, closer is the relationship between the scores. (TIF) [file pone.0126787.s002.TIF]

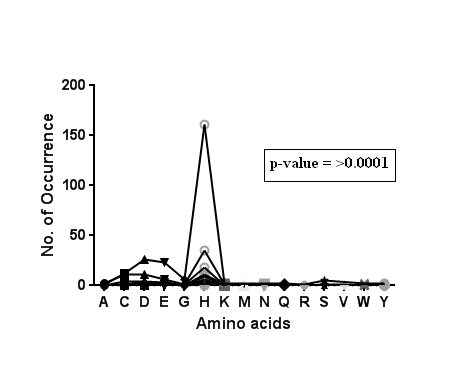

Supplement: S2 Fig — Figure represents the Statistical significance among nickel coordinating residue based on Kruskal Wallis test [p-value = >0.0001] (TIF) [file pone.0126787.s003.TIF]

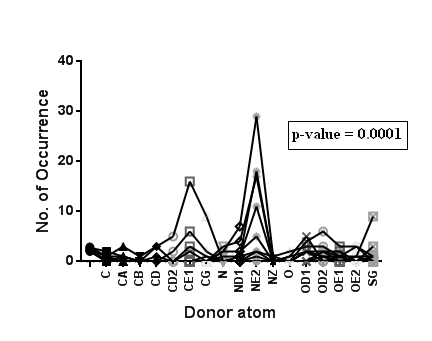

Supplement: S3 Fig — Figure represents the Statistical significance among nickel coordinating atom based on Kruskal Wallis test [p-value = 0.0001] (TIF) [file pone.0126787.s004.TIF]

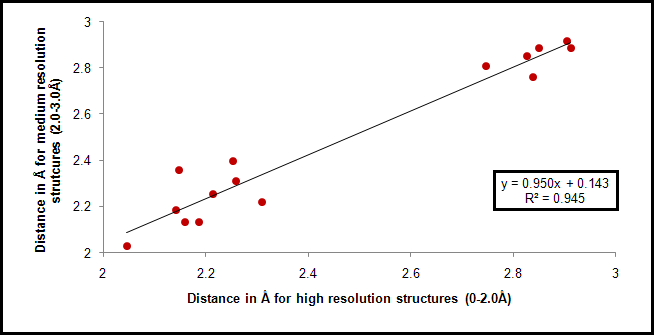

Supplement: S4 Fig — The figure shows the correlation plot for average distance of every atom type involved in nickel coordination. The R2 value given in the rectangular box indicates the correlation obtained between metal B-factor and residue B-factor. Higher the coorelation value, closer is the relationship between the scores. (TIF) [file pone.0126787.s005.TIF]

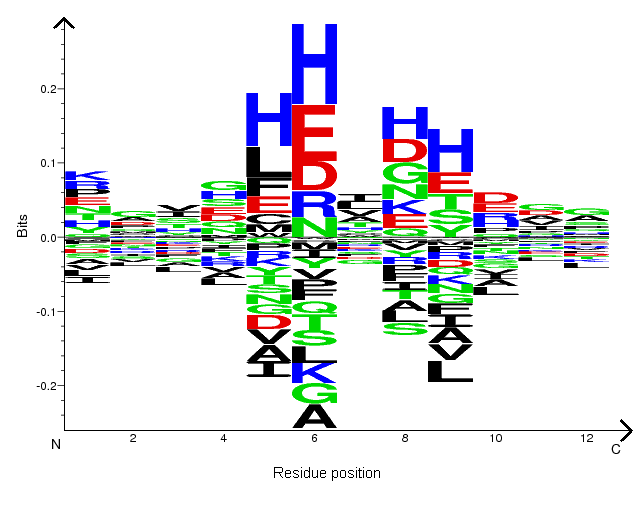

Supplement: S5 Fig — The figure shows the sequence logo for the residues within the coordination sphere. The strength of the letter indicates its predominance at the specific positions. Colors represent the nature of aminoacids where red is acidic aminoacids, blue represents basic aminoacids, green is amide residues and black is hydrophobic amino acids. (TIF) [file pone.0126787.s006.TIF]

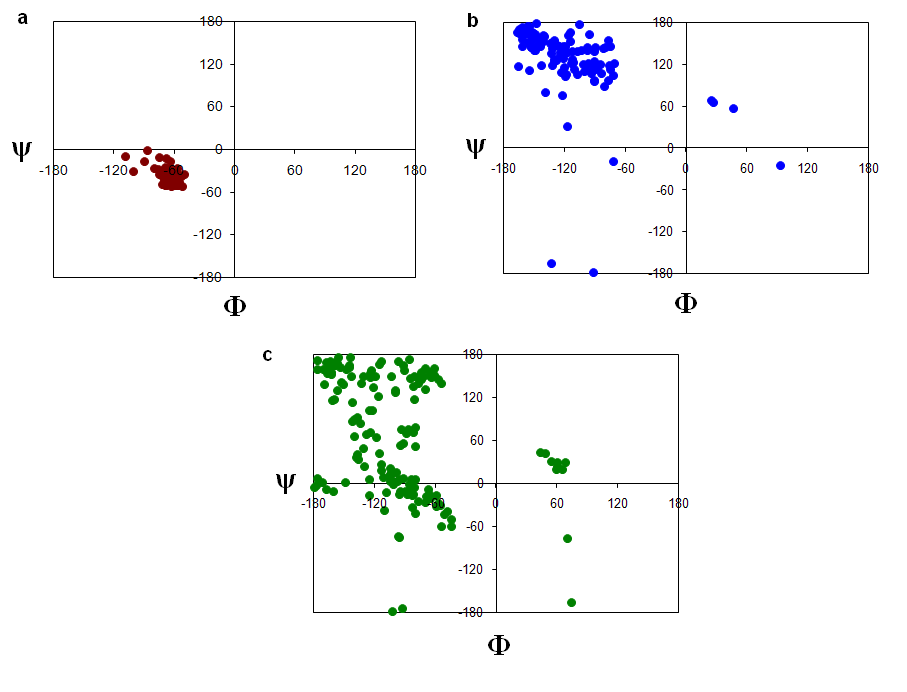

Supplement: S6 Fig — The figure represents the Ramachandran plot showing the phi (φ) and psi (ψ) angle distribution of chelate residues in a) helix b) sheet and c) turns/coils. Figure (a) shows helices to be well confined compared to sheets and coils. (TIF) [file pone.0126787.s007.TIF]

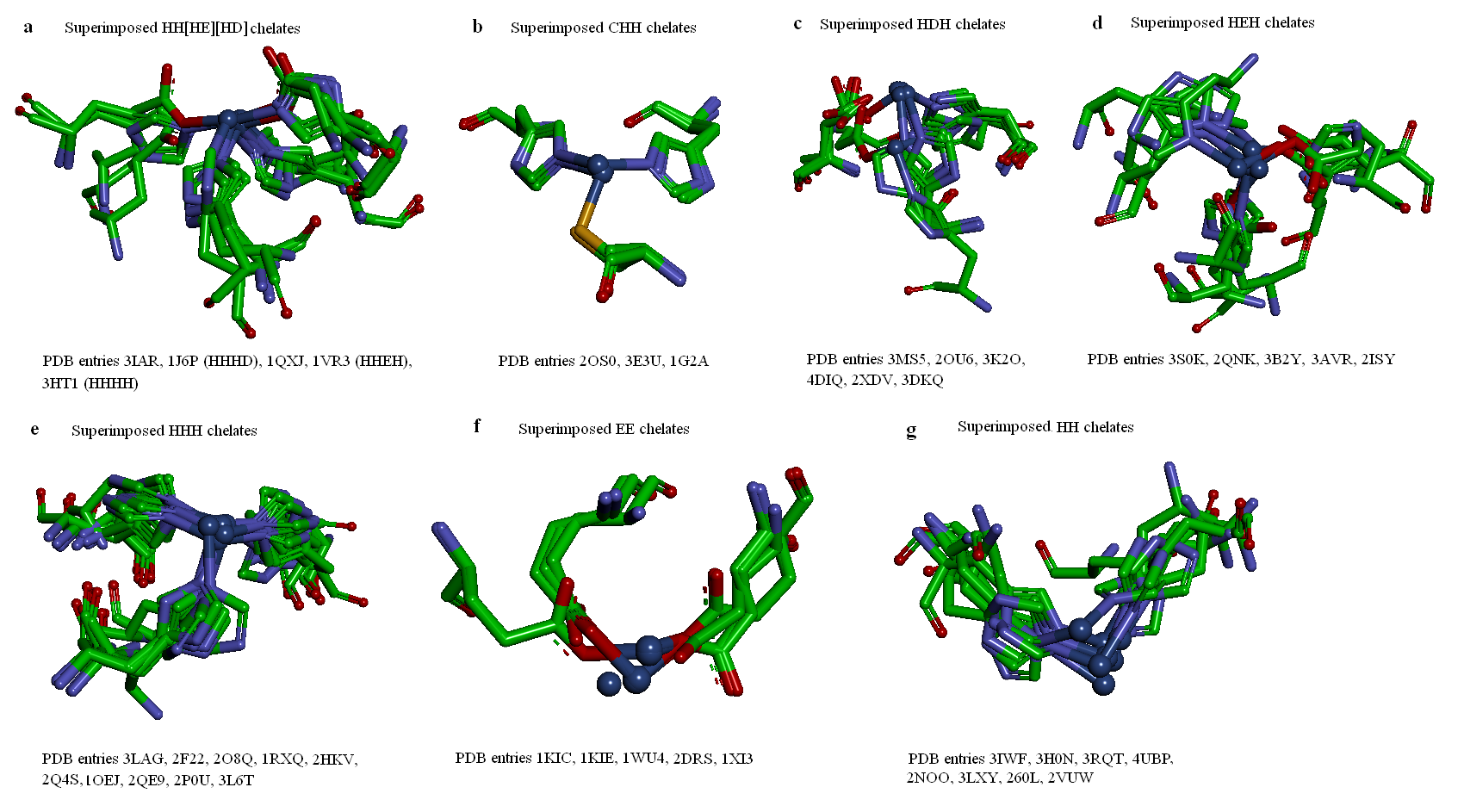

Supplement: S7 Fig — The coordinating residues are displayed as sticks and the atom coloring represents carbon (green), nitrogen (blue), oxygen (red) and sulphur (yellow). Nickel atom is shown as sphere and colored in grey. S5A Fig shows the tetra-residue chelates in distorted see-saw geometry. Fig 5b displays the trigonal geometries of tri-residue chelates, amongst which, the HHH chelate (e) shows a well demonstrated coordination. Amongst the bi-residue chelates EE (f) and HH (g), histidine based chelates display a well correlated geometry. (TIF) [file pone.0126787.s008.TIF]
